# A genome-first study of sex chromosome aneuploidies provides evidence of Y chromosome dosage effects on autism risk

**DOI:** 10.1038/s41467-024-53211-7

**Published:** 2024-10-15

**Authors:** Alexander S. F. Berry, Brenda M. Finucane, Scott M. Myers, Lauren K. Walsh, John M. Seibert, Christa Lese Martin, David H. Ledbetter, Matthew T. Oetjens

**Affiliations:** 1 Autism & Developmental Medicine Institute, Geisinger, Lewisburg, PA US; 2 Office of Research Affairs, Departments of Pediatrics and Psychiatry, University of Florida College of Medicine, Jacksonville, FL US

**Keywords:** Medical genomics, Autism spectrum disorders, Risk factors

## Abstract

A female protective effect has long been postulated as the primary explanation for the four-fold increase of autism spectrum disorder (ASD) diagnoses in males versus females. However, genetic and epidemiological investigations of this hypothesis have so far failed to explain the large difference in ASD prevalence between the sexes. To address this knowledge gap, we examined sex chromosome aneuploidy in a large ASD case-control cohort to evaluate the relationship between X and Y chromosome dosage and ASD risk. From these data, we modeled three relationships between sex chromosome dosage and ASD risk: the extra Y effect, the extra X effect, and sex chromosome haploinsufficiency. We found that the extra Y effect increased ASD risk significantly more than the extra X effect. Among females, we observed a large association between 45, X and ASD, confirming sex chromosome haploinsufficiency as a strong ASD risk factor. These results provide a framework for understanding the relationship between X and Y chromosome dosage on ASD, which may inform future research investigating genomic contributors to the observed sex difference.

## Abstract (web summary)

Genetic and epidemiological investigations have yet to reveal what drives the large difference in ASD prevalence between the sexes. Here authors examine chromosome aneuploidy in a large ASD case-control cohort and report sex chromosome haploinsufficiency is a strong ASD risk factor, while the extra Y effect increases ASD risk significantly more than the extra X effect.

## Introduction

Autism spectrum disorder (ASD) is a neurodevelopmental condition characterized by impairments in social interaction and communication, as well as restricted and repetitive patterns of behavior, interests, and activities^1^. ASD is 3.8 times more prevalent among males than females, a sex difference that has been observed consistently over time and across populations. For decades, a “female protective effect” has been endorsed as the predominant explanation for this sex ratio difference^2,3^, postulating that the risk distribution in biological females is shifted further from the ASD liability threshold than in males. Under this hypothesis, originally based on a model described in other sex-linked genetic disorders^4^, females require a larger magnitude of risk than males from genetic and environmental factors to cross the liability threshold and manifest ASD. Evidence for a female protective effect in ASD is primarily supported by epidemiological and genetic comparisons between sex-stratified cohorts, including the observation that females with ASD have a significantly greater overall burden of associated polygenic and de novo rare variants than males with ASD^5,6^.

Recently, Dougherty et al.^7^ systematically evaluated several key predictions of the liability threshold model in ASD and found several lines of evidence refuting a female protective effect^7^. They called out the need for researchers to develop an alternative conceptual framework for investigating observed ASD sex differences. The study of sex chromosome aneuploidies (SCAs), genetic conditions defined by an atypical number of X and/or Y chromosomes, provides an innovative strategy to further elucidate genomic contributors, including female protective effects, to the observed sex ratio skewing in ASD. SCAs are collectively common, with a prevalence of 1 in 450 newborns, and have well-described clinical manifestations that include congenital anomalies, hormonal imbalances, and tall or short stature. An increased prevalence of neurodevelopmental disorders, including ASD, has been well-documented through multiple SCA studies spanning several decades^8–16^. One limitation of most previous SCA studies is their reliance on data from clinically ascertained patients, potentially introducing an ascertainment bias toward those with more severe symptoms and resulting in an overestimation of ASD risk in SCA. In addition, intellectual disability (ID) and other cognitive disorders are frequent ASD comorbidities that broadly overlap with its genetic risk factors, potentially confounding the study of genetic correlations.

While the aim of many studies is to characterize the effect of a particular SCA (e.g., 47, XXY) on neurodevelopment, more comprehensive analyses of sex chromosome complements, including examinations between SCAs, can reveal contrasts in the relative contributions of X and Y gene dosage^8^. Green et al.^9^ aggregated the reported clinical prevalence of ASD among the four most common SCAs (45, X, 47, XXX, 47, XXY and 47, XYY) and proposed a model to explain the relationship between sex chromosome dosage and ASD risk^9^. The model was informed by three central hypotheses: (1) ASD risk increases with each addition of a Y chromosome, termed “the extra Y effect”; (2) ASD risk undergoes minimal to no change with each addition of an X chromosome, termed “the extra X effect”; (3) haploinsufficiency of the X chromosome increases ASD risk. Although descriptive and not yet statistically validated, this conceptualization provides a framework for using SCA research to inform the influence of both X and Y gene dosage on sex differences in ASD risk. A recent population study in Denmark by the iPSYCH consortium examined the association between SCAs and the risk of developing neuropsychiatric conditions, including ASD^10^. Although the study (here referred to as iPSYCH-SCA) broadly identified SCAs as significant risk factors for neuropsychiatric disorders, it did not examine the comparative impact of having an additional X chromosome versus an additional Y chromosome on ASD, which is crucial for assessing the validity of the Green et al. model.

Here, we created a case-control cohort (SPARKMC-SCA) to examine ASD risk among genetically-identified SCAs by combining data from the Simons Foundation Powering Autism Research (SPARK) study^17^ and Geisinger’s MyCode population cohort^18^. We leveraged this combined dataset to examine the Green et al. model of chromosome dosage and ASD risk in a statistical framework. Subsequently, we reanalyzed data from the iPSYCH-SCA study to allow for a comprehensive examination of the Green et al. model in a larger sample. With these data in hand, we performed a meta-analysis between the two studies to examine the consistency of the Green et al. model across two independent datasets. Finally, we investigated how SCAs associate with cognitive performance and social determinants of health across the UK Biobank and All of Us cohorts.

## Results

### Participants

**Fig. 1:**
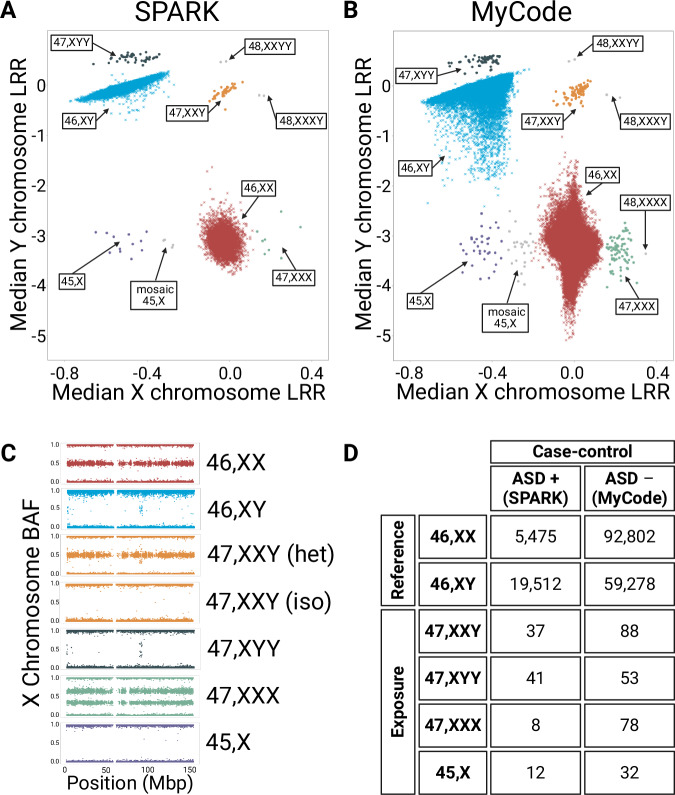
Sex chromosome aneuploidy identification in the SPARKMC-SCA cohort. **A** Log R ratio (LRR) plots are shown for the SPARK Autism Spectrum Disorder (ASD)-positive case cohort and (**B**) the MyCode ASD-negative control cohort. LRR plots in panels (**A** and **B**) are composite images that were created by juxtaposing data across genotype batches and platforms for a cohort-wide visualization. LRR plots for each individual batch are shown in Figures S1 and S2. The x-axis shows the median LRR for the X chromosome and the y-axis shows the median LRR for the Y chromosome. Colors indicate each individual’s sex chromosome complement. Participants with sex chromosome aneuploidies excluded from the analysis: 45, X; 48, XXXX; 48, XXXY; and 48, XXYY are shown in gray. **C** An example of an X chromosome-wide B allele frequency (BAF) plot for each sex chromosome aneuploidy included in the analysis. The x-axis shows the position along the X chromosome in megabase pairs (Mbp). The y-axis shows the BAF of each genotype and ranges between 0 to 1. The 47, XXY karyotype can occur by inheriting an X chromosome from each parent, called heterodisomy (het) or by inheriting homologous X chromosomes, called uniparental isodisomy (iso). **D** The table shows the number of ASD-positive cases and ASD-negative controls (outcome) with each sex chromosome complement (exposure) in the SPARKMC-SCA cohort. Source data are provided as a Source Data file.

The study population for ASD case-control analyses, the SPARKMC-SCA cohort, included 25,085 cases from SPARK (19,590 males, 5495 females) and 152,331 controls from MyCode (59,419 males, 92,912 females) (Fig. 1; Table 1; Table S1). Among the 177,416 individuals in the SPARKMC-SCA cohort, we identified 350 (98 ASD cases and 252 controls) individuals with one of the four SCAs selected for this study (Table S2). The male-to-female sex ratio among all ASD cases was 3.6:1. The male-to-female sex ratio among MyCode controls was 0.6:1, which was normalized to 1:1 for analyses (see “Methods”). The prevalence of the four SCAs among chromosomal sex-matched individuals was not significantly different from a newborn reference cohort (Figure S1), suggesting that the prevalence of SCAs among controls was representative of the general population despite the older average age of MyCode participants (56.8 years).

**Table 1:** Demographics and SCA counts

	SPARK ASD Cases	MyCode Controls
**Total counts**	25,085	152,331
**Age, mean (SD), years**	11.5 (8.9)	56.8 (18.1)
**Sex, % Female**	21.9	61.0
**Race/Ethnicity**^**a**^**, %**		
White	71.5	94.8
African	9.4	1.9
Asian	3.3	0.4
Latino	9.5	2.5
Other	3.0	0.2
Unknown	3.3	0.2
**SCA, counts (%)**		
47, XXY	37 (0.15)	88 (0.06)
47, XYY	41 (0.16)	53 (0.03)
47, XXX	8 (0.03)	78 (0.05)
45, X	12 (0.05)	32 (0.02)

*SCA* sex chromosome aneuploidy, *ASD* autism spectrum disorder, *SD* standard deviation

^a^Race/Ethnicity in SPARK is self-reported. Race in MyCode is as documented in the electronic health record.

### ASD risk among SCAs

**Fig. 2:**
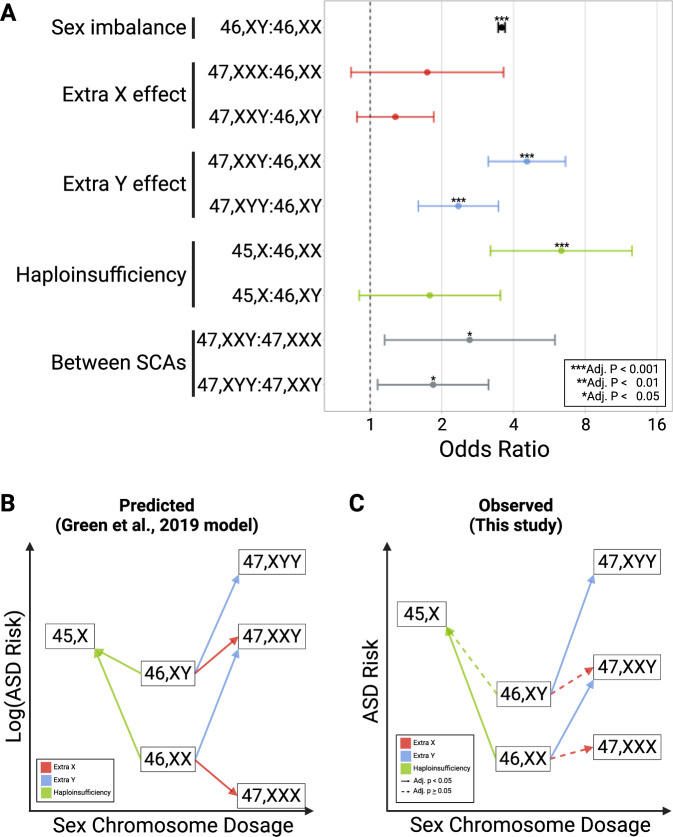
Risk of Autism Spectrum Disorder (ASD) by sex chromosome complement. The risk of ASD was compared between sex chromosome complements in the SPARKMC-SCA cohort. **A** Forest plot shows the results of logistic regression for each comparison. Points denote calculated odds ratio and error bars represent 95% confidence intervals. The groupings on the *Y*-axis and colors indicate the central hypothesis from Green et al.^9^, tested in the comparison. Asterisks denote level of statistical significance for each 2-sided test, adjusted using Benjamini-Hochberg false discovery rate correction (Adj. p). Adjusted *p*-values for each comparison are as follows: 46, XY:46, XX, < 2.22 × 10^−16^; 47, XXX:46, XX, 0.18; 47, XXY:46, XY, 0.23; 47, XXY:46, XX, 9.2 × 10^−15^; 47, XYY:46, XY, 5.1 × 10^−5^; 45, X:46, XX, 4.9 × 10^−7^; 45, X:46, XY, 0.14; 47, XXY:47, XXX, 0.04; 47, XYY:47, XXY, 0.04. Sample sizes used to derive statistics for each sex chromosome complement are as follows: 46, XX, 98,277; 46, XY, 78,790; 47, XXY, 125; 47, XYY, 94; 47, XXX, 86; 45, X, 44. **B** Representation of the effects of sex chromosome dosage on autism risk in those with a sex chromosome aneuploidy relative to those with two sex chromosomes as shown in the Green et al. model which is based on the autism prevalence reported by clinical studies of sex chromosome aneuploidies. ASD risk is log-transformed to emphasize a consistent pattern between the Green et al. model and the observed results. **C** The plot summarizes the results of logistic regression analyses performed here in a large ASD case-control cohort. Solid lines indicate the association is statistically significant at adjusted *P* < 0.05 while dashed lines indicate adjusted *P* ≥ 0.05. All tests were 2-sided. *P*-values were not reported in the Green et al. model. Source data are provided as a Source Data file.

In our confirmatory analysis of the Green et al. model, we examined the association between SCAs and ASD risk to test the effect of an extra X, the effect of an extra Y, and the effect of sex chromosome haploinsufficiency in the SPARKMC-SCA cohort (Fig. 2, Figure S2, Figure S3). First, we tested the extra X effect by modeling the association between a supernumerary X chromosome and ASD risk in analyses of 47, XXX and 47, XXY relative to 46, XX and 46, XY, respectively. In these models, 47, XXX was not significantly more likely to be associated with ASD compared to 46, XX, and 47, XXY was not significantly more likely to be associated with ASD compared to 46, XY (adjusted *p* ≥ 0.05). We note that the 95% confidence interval (CI) for the 47, XXX comparison is wide and ranges from 0.83–3.63 (Fig. 2).

Second, we tested the extra Y effect by modeling the association between a supernumerary Y chromosome and ASD risk in analyses of 47, XXY and 47, XYY relative to 46, XX and 46, XY, respectively. 47, XXY was significantly more likely to be associated with ASD compared to 46, XX (odds ratio (OR), 4.6; 95% CI, 3.1–6.6), and 47, XYY was significantly more likely to be associated with ASD compared to 46, XY (OR, 2.4; 95% CI, 1.6–3.5) (Fig. 2).

Third, we tested the effect of haploinsufficiency by modeling associations between ASD risk in 45, X relative to 46, XX and 46, XY. 45, X was significantly more likely to be associated with ASD compared to 46, XX (OR, 6.4; 95% CI, 3.2–12.6), but not compared to 46, XY (adjusted *P* > 0.05) (Fig. 2). When defining non-mosaic 45, X using an 80% non-mosaicism threshold, the association between ASD risk in 45, X relative to 46, XX (OR, 6.8; 95% CI, 2.6–18.0) was nearly identical to criteria requiring 60% non-mosaicism for 45, X) (Table S3), suggesting that the association between 45, X and ASD is robust to the level of mosaicism.

The central hypotheses of the Green et al. model were also supported by analyses performed between SCA groups. 47, XXY was significantly more likely to be associated with ASD compared to 47, XXX (OR, 2.7; 95% CI, 1.2-6.0). 47, XYY was significantly more likely to have ASD compared to 47, XXY (OR, 1.8; 95% CI, 1.1-3.1). The impact of SCAs in contexts useful for clinical prognostication and assessment of the epidemiological impact, such as penetrance and population attributable fraction, respectively, are shown in the supplement (Supplemental Methods, Table S4).

### Meta-analysis of SPARKMC-SCA and iPSYCH-SCA

We examined whether a meta-analysis incorporating data from the iPSYCH-SCA cohort would strengthen the evidence of a stronger extra Y effect on ASD risk relative to the extra X effect. As a first step, we re-analyzed data from the iPSYCH-SCA cohort to generate risk estimates that were not reported in the initial publication. Specifically, we modeled comparisons between groups with different chromosomal sexes (e.g., 47, XXY vs. 46, XX) and comparisons between SCAs (e.g., 47, XXY vs. 47, XYY) (Table S5). Additionally, as the iPSYCH-SCA publication reported hazard ratios as risk estimates between SCAs and sex matched controls, we calculated ORs from the reported SCA sample sizes for the purpose of the meta-analysis. As expected, the ORs calculated in the iPSYCH-SCA study closely matched the hazard ratios reported in the original publication. With the iPSYCH-SCA estimates in hand, the total sample included in the meta-analysis incorporated a total of 565 individuals with an SCA among 47,285 cases and 194,985 control subjects.

**Fig. 3:**
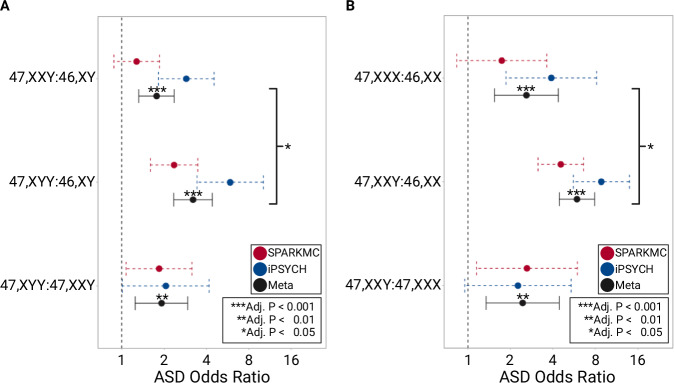
Meta-analysis of SPARKMC-SCA and iPSYCH-SCA. The ASD risk estimates from the SPARKMC-SCA and iPSYCH-SCA studies were meta-analyzed for each comparison shown. **A** Forest plot shows ASD risk associated with 47,XXY or 47,XYY relative to a 46,XY reference. **B** Forest plot shows ASD risk associated with 47,XXX or 47,XXY relative to a 46,XX reference. Brackets indicate the results of comparisons between extra Y and extra X effect sizes from the meta-analysis. Colors identify whether the odds ratio resulted from analyses from the SPARKMC-SCA cohort alone (red), the iPSYCH-SCA cohort alone (blue), or from meta-analyzing both results together (black). Red and blue points denote odds ratio calculated from logistic regression analyses and error bars represent the associated 95% confidence intervals. Black points denote effect sizes calculated from each meta-analysis (Meta) using a fixed-effects model in metafor, and error bars represent the associated 95% confidence intervals. Asterisks denote level of statistical significance for each 2-sided test, adjusted using Benjamini-Hochberg false discovery rate correction (Adj. p), and are shown only for meta-analysis results. Adjusted p-values for all comparisons are shown in Tables S9 and S10 in the Supplement. Sample sizes used to derive statistics for each sex chromosome complement in the SPARKMC-SCA cohort are as follows: 46, XX, 98,277; 46, XY, 78,790; 47, XXY, 125; 47, XYY, 94; 47, XXX, 86. Sample sizes used to derive statistics for each sex chromosome complement in the iPSYCH-SCA cohort are as follows: 46, XX, 26,524; 46, XY, 38,114; 47, XXY, 87; 47, XYY, 89; 47, XXX, 28. Source data are provided as a Source Data file.

In the meta-analysis, we first compared ASD risk associated with 47, XYY and 47, XXY with respect to 46, XY as the reference group. We observed that both 47, XYY (OR, 3.2; 95% CI, 2.3–4.4) and 47, XXY (OR, 1.8; 95% CI, 1.3–2.4) were associated with increased ASD risk (Fig. 3A, Table S6). By comparing these two risk estimates, we found that the effect of 47, XYY was significantly larger compared to the effect of 47, XXY (Adj. *P* < 0.05). Next, we compared the risk of ASD among 47, XXY and 47, XXX with respect to 46, XX as the reference group. We observed that both 47, XXY (OR, 5.9; 95% CI, 4.4-7.9) and 47, XXX (OR, 2.6; 95% CI, 1.5–4.4) were associated with an increase in ASD risk (Fig. 3B). The risk estimate associated with 47, XXY was significantly larger than 47, XXX (Adj. *P* < 0.05). Lastly, between SCA analyses were consistent with a larger extra Y effect as individuals with 47, XYY had increased ASD risk compared to 47, XXY (OR, 1.9; 95% CI, 1.2–2.9). Similarly, 47, XXY was significantly more likely to be associated with ASD compared to 47, XXX (OR, 2.4; 95% CI, 1.3–4.4).

### Prevalence of SCAs Across Cohorts

**Fig. 4:**
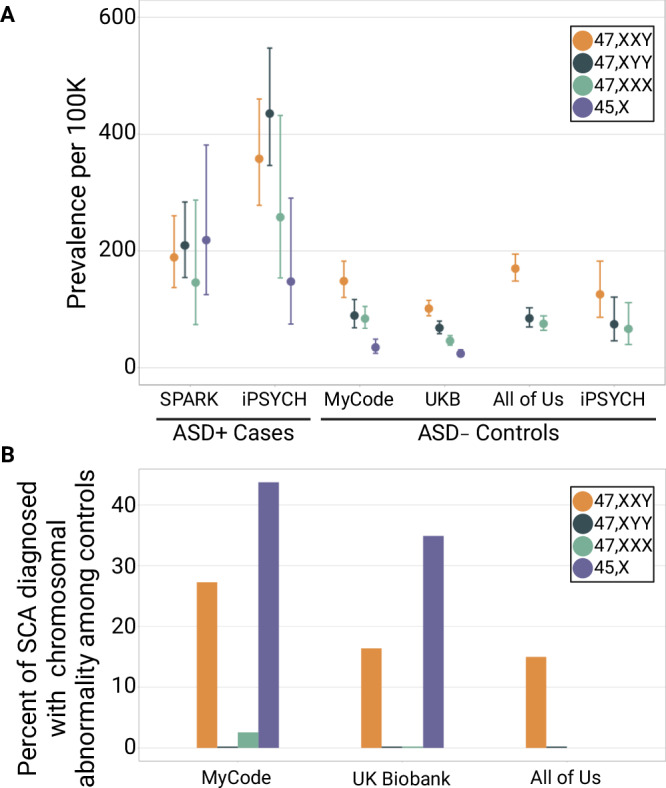
Prevalence and diagnosis rate of sex chromosome aneuploidies (SCAs) across cohorts. **A** Forest plot shows the prevalence of each sex chromosome aneuploidy among Autism Spectrum Disorder (ASD)-positive cases from SPARK (*n* = 25,085) and iPSYCH (*n* = 22,200), and among ASD-negative controls from MyCode (*n* = 152,331), UK Biobank (n = 487,865), All of Us (*n* = 308,248), and iPSYCH (*n* = 42,654). Error bars represent 95% confidence intervals for binomial probabilities. **B** Bar plot shows the percent of individuals with a genetically-identified SCA who have a clinical diagnosis for a chromosomal abnormality among ASD-negative controls. SCA diagnosis data was not available for SPARK or iPSYCH participants. For visualization, a value of 0.25% in the plot corresponds to an actual percent diagnosed of 0%. 45, X prevalence and diagnosis rates are not shown for the All of Us cohort or for iPSYCH controls, and 47, XXX diagnosis rates are not shown for the All of Us cohort because no data or statistics can be reported that allow a participant count of 1 to 20 (All of Us) or 1 to 5 (iPSYCH) to be derived. Source data are provided as a Source Data file.

Among newborn males, the reported incidence of 47, XXY ranges from 1:500 to 1:650, while the incidence of 47, XYY is reported to be 1:1000^19,20^. The incidence of 47, XXY is expected to be between 1.5 to 2 times as high as 47, XYY, as it can arise from nondisjunction at meiosis I in the male or either division in the female. By contrast, 47, XYY can only arise in the male from nondisjunction of a Y chromosome during meiosis II. We hypothesized that the relative associations between the extra X and extra Y effects with ASD would distort this ratio between 47, XXY and 47, XYY among males in ASD cohorts relative to population cohorts. Consistent with this expectation, when we calculated the prevalence of SCAs in four population cohorts we found that the 47, XXY-to-47, XYY ratio was similar across population biobanks and ranged between 1.49-fold to 2.01-fold (Fig. 4A). We also observed that the frequency of an SCA clinical diagnosis was similar across population biobanks (Fig. 4B). Most individuals with a genetically-identified SCA do not have a clinical SCA diagnosis. In contrast to population biobanks and control samples, the 47, XYY-to-47, XXY ratio among ASD cases was 0.9 in SPARK (47, XYY: 209 per 100 K; 47, XXY: 189 per 100 K) and 0.8 in iPSYCH (47, XYY: 435 per 100 K; 47, XXY: 358 per 100 K). These observations are consistent with the extra Y effect skewing of the relative frequencies of 47, XXY and 47, XYY in populations of ASD probands relative to population cohorts.

### Cognitive Performance and Social Determinants of Health

**Fig. 5:**
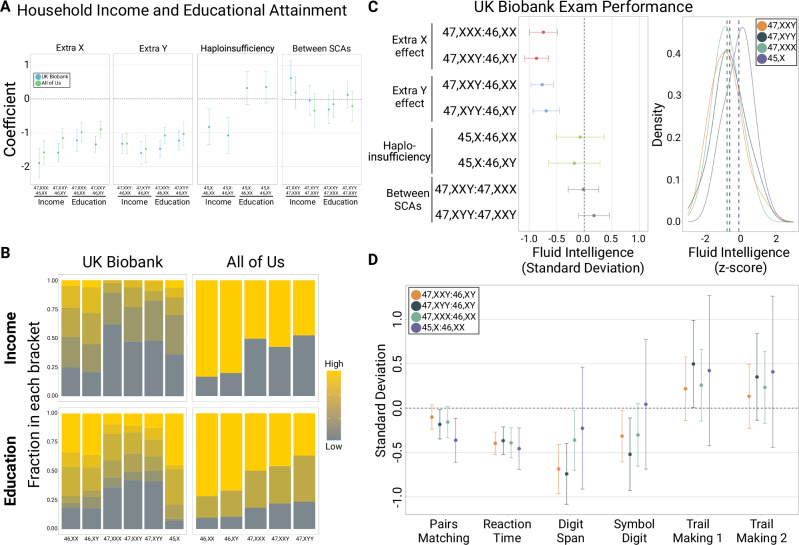
Household income, educational attainment, and cognitive measurements associated with sex chromosome aneuploidy (SCA). **A** Forest plots show the results of ordinal regression analyses of the correlation between SCA and household income or educational attainment. Error bars indicate 95% confidence intervals. Household income and educational attainment data were only available for the UK Biobank (blue) and All of Us (green) ASD-negative control cohorts. **B** Stacked bar plots show the proportion of each SCA in each household income or educational attainment bracket in the UK Biobank and All of Us ASD-negative control cohorts. For the All of Us cohort, no data or statistics can be reported that allow a participant count of 1 to 20 to be derived, therefore only two household income brackets are shown (<$10 K per year and ≥ $10 K per year), and three educational attainment brackets are shown (did not complete 12^th^ grade, completed 12^th^ grade or GED, and completed at least one year beyond 12^th^ grade). Bracket colors range from gray for the lowest household income or educational attainment bracket to yellow for highest household income or educational attainment bracket. **C** Fluid intelligence exam performance in the UK Biobank was measured by an exam given to participants. Forest plot shows the standard deviation and 95% confidence intervals of fluid intelligence exam performance for each comparison between sex chromosome complements, calculated using linear regression. Colors indicate the central hypothesis from Green et al.^9^, tested in the comparison. The density plot to the right shows the distribution of fluid intelligence exam performance z-scores for each SCA. Vertical dashed lines denote the mean fluid intelligence exam performance z-score for each SCA. Sample sizes used to derive statistics for fluid intelligence exam performance by sex chromosome complement are as follows: 46, XX, 128,942; 46, XY, 106,783; 47, XXY, 87; 47, XYY, 72; 47, XXX, 56; 45, X, 19. D) Forest plot shows the standard deviation (points) and 95% confidence intervals (error bars) of six additional cognitive tests (pairs matching (*n* = 472,378), reaction time (*n* = 482,486), digit span (*n* = 161,626), symbol digit (*n* = 131,797), numeric trail making (*n* = 119,731), and alphanumeric trail making (*n* = 119,131)) for sex-matched comparisons between SCAs and 46, XX or 46, XY, calculated using linear regression. Source data are provided as a Source Data file.

As cognition and ASD share many genetic underpinnings, we hypothesized that the pattern of association observed between SCAs and ASD would be similar across measures of cognitive performance and social determinants of health, including measures of academic- and career-based achievement. Alternatively, a departure from this pattern would suggest that the association between sex chromosome dosage and ASD acts at least partially independently of a deleterious effect on cognition. We examined associations between SCA, household income, educational attainment, and seven measures of cognitive performance among nearly 800,000 individuals from two population cohorts: the UK Biobank (UKB; *n* = 487,865) and All of Us (*n* = 308,248) (Fig. 5). In these analyses, we found that 47, XXY, 47, XYY, and 47, XXX all had significantly lower household income and educational attainment compared to 46, XX and 46, XY. With the exception of a modest association between household income in 47, XXY relative to 47, XXX in the UK Biobank, the effect sizes between supernumerary SCAs were not significantly different from one another (*p* > 0.05) and do not support a difference between the extra X or extra Y effect on these measures. In contrast, 45, X did not have significantly different fluid intelligence exam performance or educational attainment from 46, XX or 46, XY despite its large effect on ASD risk observed in the SPARKMC-SCA and iPSYCH-SCA studies. Together, these results suggest that the pattern of association between sex chromosome dosage and cognitive performance and social determinants of health is not the same as ASD.

### Sensitivity analyses

To evaluate how underdiagnosis of ASD in control cohorts could affect the relationship between SCA and ASD, the associations were re-calculated using simulated control populations by varying 1) the prevalence of undiagnosed ASD and 2) the enrichment of SCAs among controls with undiagnosed ASD. Importantly, the resulting ORs of these simulations all fall within the confidence intervals calculated using the MyCode control cohort with ASD and ID diagnoses removed (Figure S4). Relative to the simulated ORs, the ORs of the extra X, extra Y, and haploinsufficiency effects in this report are smaller, and therefore, likely conservative estimates of ASD risk. To test the impact of removing ID diagnoses from the MyCode control cohort, analyses of sex chromosome dosage and ASD were repeated using controls where only participants with an ASD diagnosis were removed. The results were consistent whether or not ID diagnoses were removed from the control cohort (Table S7).

We conducted a sensitivity analysis to examine the potential for bias in the ASD diagnosis threshold among SCAs, which could influence their inclusion and prevalence in the SPARK cohort. If a bias in the ASD diagnostic criteria for SCAs exists, it is anticipated that this group would also exhibit differences in the severity of ASD symptoms compared to the broader ASD population. To test for evidence of differences in a diagnostic bias, we compared measures of ASD severity between those with and without an SCA in the SPARK cohort. We found that mean Social Communication Questionnaire (SCQ) and Vineland Adaptive Behavior Scales (VABS) Adaptive Behavior Composite (ABC) scores did not differ significantly between those with an SCA and chromosomal sex-matched participants without an SCA and fall within the expected range for an ASD cohort (Figure S5)^13,14,21^. Additionally, among ASD cases, those with an SCA had a similar age of initial ASD diagnosis, and parental age at birth compared to those without an SCA. These results suggest that individuals in SPARK with an SCA are not different from the remainder of the SPARK cohort with respect to ASD severity.

Lastly, as a sensitivity analysis to test the robustness of associations regardless of the source of samples for the control cohort, four additional case-control cohorts were created by combining ASD cases from the SPARK cohort and controls from (1) All of Us, (2) the UKB, (3) siblings of SPARK probands, and (4) a newborn reference cohort^19^ (Table S1). We examined the extra X effect, the extra Y effect, and the effect of sex chromosome haploinsufficiency on ASD in each cohort (Table S8). The results broadly were consistent across cohorts with the exception of a few associations between 47, XXY, 47, XXX and ASD only observed when using the UK Biobank as controls. The associations between sex chromosome complement and ASD risk, cognitive performance and social determinants of health were consistent when analyses were restricted to participants with European ancestry (Table S9, Table S10, Table S11).

## Discussion

In this study, we examined four SCAs in the SPARKMC-SCA cohort to explore how variations in sex chromosome dosage impact ASD risk. In our primary analysis examining the association between SCAs and ASD, we found the extra Y effect was significantly larger than the extra X effect. This conclusion was drawn from our observation that individuals with 47, XYY showed a 2.4-fold higher risk of ASD compared to those with 46,XY, and supported by our observation that individuals with 47, XXY were at a 4.6-fold higher risk compared to those with 46,XX. The 45,X sex chromosome complement was associated with a 6.4-fold increase in ASD risk relative to 46,XX, confirming sex chromosome haploinsufficiency as a strong ASD risk factor. We did not, however, detect an association between the extra X effect and ASD risk in the analysis of the SPARKMC-SCA cohort.

A larger extra Y effect was further supported when we meta-analyzed results from the SPARKMC-SCA and iPSYCH-SCA studies. In these analyses, this observation was consistent whether the additional sex chromosome was identified in a chromosomal male or female individual. We found that individuals with 47, XYY were at double (OR, 1.9, 95% CI, 1.2–2.9) the risk of ASD compared to 47, XXY and this result became more statistically significant upon meta-analysis compared to either study alone (Adj. P_meta_ = 0.0048; Adj. P_SPARKMC-SCA_ = 0.033; Adj. P_iPSYCH-SCA_ = 0.056). Similarly, we found that individuals with 47, XXY were again double (OR = 2.4, 95% CI, 1.3–4.4) the risk of ASD compared to 47, XXX and again statistical significance increased in the meta-analysis (Adj. P_meta_ = 0.0050; Adj. P_SPARKMC-SCA_ = 0.030; Adj. P_iPSYCH-SCA_ = 0.073). In these SCA analyses, very little heterogeneity was detected in the effect size between the studies (i^2^ < 0.01).

Overall, the pattern of risk between sex chromosome dosage and ASD largely agreed with those reported by the iPSYCH study. The rank order of the effect size in sex-matched comparisons was consistent across studies: 45, X had the largest effect on ASD risk, followed by 47, XYY, 47, XXY, and 47, XXX. However, significant heterogeneity was identified in the magnitude of the effect sizes, which may be explained by how the ASD cases were ascertained. Most ASD cases in SPARK are U.S. children with a documented clinical ASD diagnosis whose parents volunteered them to participate, while ASD cases in iPSYCH were ascertained from the Danish Psychiatric Central Research Register, which contains data on all admissions to Danish psychiatric in-patient facilities. Ascertainment differences between the two approaches may have led to variations in the patient groups, such as the presence of ID, the age at ASD diagnosis, and other factors that could lead to differences in the prevalence of SCAs. Underdiagnosis of ASD in our adult control cohort may also explain some of the differences in effect size between our study and the iPSYCH study. Our simulation of control populations under different prevalence of undiagnosed ASD showed that the results of our sex-matched analyses are likely to be a conservative estimate of ASD risk associated with SCA.

Sex significantly impacts ASD risk without having a major influence on cognitive performance. In other words, the increased rates of ASD among males cannot be explained by cognitive differences between the sexes. If the extra Y effect is mediated through cognitive performance, we would expect a larger, deleterious effect on these measures relative to the extra X effect. However, our analyses found that both an extra X and Y chromosome conferred similar effects on cognitive performance. This suggests a pattern consistent with the relationship between sex, ASD, and cognitive performance: the difference between the effect of increased X and Y chromosome dosage on ASD risk was not explained by their relationships with cognitive performance. Furthermore, we observed that 45,X conferred the least impact on cognitive performance despite having the largest effect on ASD risk in both the SPARKMC-SCA (OR = 6.4) and iPSYCH-SCA studies (hazard ratio (HR) = 8.45).

The larger extra Y effect on ASD risk suggests that one or more dosage sensitive genes on the Y chromosome contributes to males’ heightened ASD susceptibility. The Y chromosome is the smallest chromosome and contains roughly 23 Mb of unique sequence, termed the male-specific region (MSY)^22^. Since the divergence of the X and Y chromosomes over 200 million years ago, 18 genes on the MSY have maintained a strong homology with an X chromosome counterpart across mammalian species. These genes, called gametologs, have diverse expression profiles across adult tissues, including the brain^23^. Similar to the many ASD genes reported in the literature, the gametologs have strong evidence of purifying selection and dosage sensitivity, which is thought to underscore their importance to human health and development^24–26^. Two gametologs, *NLGN4Y* and *USP9Y*, have been proposed as candidate ASD loci^27^.

Recent research has identified an abundance of changes in gene expression across the autosomes and sex chromosomes that occur in the presence of an SCA^28^. The extra Y effect on ASD risk could be a consequence of a supernumerary Y chromosome’s impacts on global gene expression. For example, a pair of gametologs, *ZFX* and *ZFY*, transcription factors on the X and Y chromosomes respectively, were identified as the regulators of the hundreds of genes found to have altered expression in the presence of an SCA^29–31^. Importantly, the effect of *ZFY* is sensitive to changes in Y dosage. Intriguingly, variants in *ZFX* were recently identified as the cause of an X-linked ID syndrome^32^, which demonstrates the importance of this gene to neurodevelopment. While the phenotypic consequences of variants in its Y-linked gametolog, *ZFY*, are yet to be reported, this gene is a candidate mediator of the extra Y effect.

The female protective effect has long been speculated to be mediated by protective factors on the second X chromosome among 46, XX females; however, no genetic factor has been definitively pinpointed. In fact, the theory that females are more resistant to ASD risk factors has come under scrutiny. For example, a comprehensive family-based study, involving nearly 1 million children from the Swedish National Patient Register, examined whether females without ASD who have siblings with ASD possess a higher genetic predisposition for autism than their male counterparts^33^. The female protective effect theory predicts that the offspring of unaffected female siblings of ASD probands would have a higher ASD risk compared to the offspring of unaffected male siblings. However, the Swedish family-based study concluded that the difference in ASD prevalence among offspring from the two risk groups was not sufficient to explain the observed sex ratio difference in ASD. Our data and the Swedish family-based study provide evidence that the underlying biology of the sex imbalance in ASD is more complex than what can be fully encompassed by the current theory of the female protective effect. Further studies are needed to demonstrate the biological mechanism that underlies ASD risk associated with supernumerary Y chromosome dosage and if it relates to the sex difference in ASD.

This study has several limitations. First, the different cohorts from which cases and controls were derived may have different ascertainment biases because parents of pediatric ASD cases enroll their children in research, while adults included in population cohorts independently volunteer, likely excluding more severe ASD presentations. Second, SCQ and VABS data were not available for control participants in either cohort and were solely defined by the lack of an ASD diagnosis, either in the electronic health records (EHR) or by self- or parental-report. Third, we were unable to test for associations between hormonal differences associated with SCA and ASD risk. While 47, XXY is associated with a different hormonal environment compared to 46, XY and 47, XYY, our study found no difference in ASD rate between 47, XXY and 46, XY. Additionally, there is no evidence that 47, XYY has a different hormonal profile than 46, XY, but our results show a large difference in ASD risk. Finally, many hormonal differences occur post-puberty, and SPARK participants are largely diagnosed before puberty. Therefore, hormonal differences are unlikely to explain the association between ASD and Y chromosome dosage found here. Lastly, in the primary analysis of ASD, the sample was not restricted to a specific race or ethnic group. Sensitivity analyses using controls from All of Us, a racially and ethnically diverse cohort, showed that ancestry alone does not impact the results. Additional sensitivity analyses using the subset of each cohort with European ancestry yielded results consistent with primary analyses. However, the impact of ancestry on the ascertainment of genetic ancestry groups into ASD cohorts, such as SPARK, is not well described and could not be accounted for in our statistical modeling.

This study bridges findings from decades of observational studies of SCA and ASD among clinical cohorts with emerging data from large-scale genetic biobanks. Studies in genetic biobanks, such as this, continue to demonstrate that SCAs are significantly under-ascertained by medical providers. We demonstrate that genome-first studies of SCAs are a powerful tool for modeling the relationship between sex chromosome dosage and phenotypic traits. While biological sex is a known correlate for many medically important outcomes related to cardiovascular disease, autoimmune conditions, neuropsychiatric disorders, and others, the contribution of sex chromosomes to observed sex differences is generally not well understood. While we focused here on ASD, this study provides a broader framework for using SCA research to understand the role of X and Y dosage on other clinical phenotypes with unequal sex ratios.

## Methods

### Study participants

We created an ASD case-control dataset by combining phenotypic and genomic data from two separate cohorts. ASD cases were collected from the SPARK study^34^, which includes U.S.-based probands with an ASD diagnosis. All participants were recruited to SPARK under a centralized Institutional Review Board (IRB) protocol (Western IRB Protocol #20151664) and provided written informed consent to take part in the study. Controls were collected from Geisinger’s MyCode Community Health Initiative, a large U.S. health care cohort that primarily recruits adult participants^18,35^. Informed consent was obtained from adult patients and from the parents or guardians of pediatric patients. The Geisinger IRB approved the study. These two cohorts were combined to form the ASD case-control cohort, SPARKMC-SCA, used in the primary analyses here. Two additional large epidemiological control populations were used in sensitivity analyses: the U.S.-based All of Us cohort^36^; and the UK-based UKB cohort^37^. Informed consent for all All of Us participants was conducted in person or through an eConsent platform that includes primary consent, HIPAA Authorization for Research use of EHRs and other external health data, and Consent for Return of Genomic Results. The protocol was reviewed by the All of Us IRB. The UKB has ethical approval from the North West Multi-Centre Ethics Committee. UKB data was accessed under project 49945. All UKB participants provided informed consent to participate in UK Biobank projects.

Linked EHR and genotype array data were available from MyCode, All of Us, and UKB participants. ASD, ID, and chromosomal abnormality diagnoses were identified from linked EHR records in MyCode, All of Us, and the UKB using International Classification of Disease (ICD) codes (Table S12). MyCode, All of Us, and UKB participants with ASD or ID were removed to curate control cohorts, which secondarily eliminated overlap between SPARK participants and controls from MyCode and All of Us (Table S12; Table S13). Although ASD and ID are frequently comorbid^38,39^, the EHRs of dually-affected adults rarely document both diagnoses; we therefore excluded controls with either ASD or ID diagnosis codes to ensure an ASD-negative cohort. In addition to controls identified in population cohorts, 3683 ASD-negative siblings available from the SPARK cohort were used as an additional control group for comparisons with ASD-positive groups. A reference cohort containing 34,904 newborns with sex chromosome examinations was used as an additional control cohort^19^. We note that SPARK siblings were not evaluated for ID and individuals in the newborn reference cohort were not evaluated for ASD or ID. Race/ethnicity in SPARK is self-reported. Race/ethnicity in MyCode is as documented in the EHR. In primary analyses, inclusion of samples was not restricted to a specific race or ethnic group.

A recent study evaluated sex-matched associations between SCA and ASD using the Danish iPSYCH cohort^10^. The iPSYCH cohort includes individuals born in Denmark between 1981 and 2008. ASD cases were defined as having an ASD diagnosis registered in the Danish Psychiatric Central Research Register. The counts of ASD-positive cases and ASD-negative controls by sex chromosome complement in the iPSYCH cohort were derived from this study. We excluded 45, X from re-analyses of the iPSYCH-SCA study because counts of ASD-negative participants with 45, X could not be reported in iPSYCH-SCA to prevent deduction of counts below five.

### Identification of SCA

X and Y chromosome dosage was determined in SPARK and MyCode using an array-based approach (Figure S6, Figure S7, Figure S8)^35^. The median log R ratio of the genotype probes across the X chromosome (LRRx) was plotted against the median log R ratio of the Y chromosome (LRRy) for each genotype array platform. The sex chromosome complement of each participant was determined by the LRRy:LRRx ratio relative to thresholds created for each cohort and genotype platform (Table S14). Among individuals with 45, X, the percent mosaicism (i.e., the proportion of cells with the 45, X complement) was estimated by dividing the median LRRx by the lowest observed female value within each sequencing platform. Individuals were included in the 45, X SCA group if at least an estimated 60% of their cell line was 45,X^35,40^. The remaining individuals with evidence of 45, X but a smaller percentage of 45, X in their cell line were removed from downstream analyses. LRR and B allele frequency distributions across the entire X and Y chromosomes were manually assessed for each SCA to confirm each call. Chromosomal sex was defined by the presence (male) or absence (female) of a Y chromosome. Individuals whose chromosomal sex did not match self-reported sex were removed (Table S15).

SCA identification in the All of Us and UKB cohorts was similarly performed using genotype arrays and is described separately in the supplement (Supplemental methods). We note here that analysis of SCAs in All of Us is restricted to groups ≥20. As fewer than 20 participants with 45, X were identified in the All of Us cohort, we did not evaluate the effect of sex chromosome haploinsufficiency in this cohort.

### Phenotype data

To compare ASD-related phenotypes by sex chromosome complement, we collected parent-reported data from the SCQ, a clinical tool used to screen patients for ASD; the VABS; proband age at ASD diagnosis; and parental age of SPARK participants. Nonverbal or minimally verbal SPARK participants were excluded from the SCQ analyses due to their small number. Differences in household income and educational attainment were compared by sex chromosome complement in the UK Biobank and All of Us cohorts.

Data from the UK Biobank and All of Us were used to compare cognitive performance and social determinants of health by sex chromosome complement. UK Biobank participants performed cognitive exams including Fluid Intelligence, Pairs Matching, Reaction Time, Digit Span, Symbol Digit, Numeric Trail Making, and Alphanumeric Trail Making. Educational attainment and household income data were available for UK Biobank participants as part of the initial assessment exam and were available for All of Us participants as part of The Basics survey offered to all participants. Transformations of cognitive test results and educational attainment followed Kendall et al.^41^ and are detailed in the Supplemental Methods. Educational attainment in the UK Biobank was grouped into six brackets based on highest qualification obtained. Participants who responded “other professional qualifications” were excluded. Annual household income in the UK Biobank was grouped into five brackets. Educational attainment in All of Us was grouped into eight brackets based on highest grade completed. Annual household income in All of Us was grouped into nine brackets.

### Statistical Methods

To model the association between SCA and ASD, a dataset of counts per sex chromosome complement among the ASD cases from SPARK and controls from MyCode was created. The four most common SCA were selected for analysis: 45, X; 47, XXY; 47, XYY; and 47, XXX. Individuals with four or more sex chromosomes were excluded from further analysis due to small sample sizes. ORs for having an ASD diagnosis in those with each of the four SCA (1) relative to 46, XX, (2) relative to 46, XY, and (3) between SCAs were calculated using logistic regression. Logistic regression analyses were repeated using each control cohort (All of Us, UKB, SPARK siblings, newborn reference cohort^19^), in place of MyCode controls to determine whether the results were sensitive to which cohort was used for controls. All P values were adjusted using Benjamini-Hochberg false discovery rate correction^42^. Adjusted P  <  .05 was considered significant. All reported P values are 2-sided.

The sex ratios in MyCode, All of Us, and the UKB are female-skewed due to sex differences in health care utilization and willingness to participate in research and are not representative of the general population. Therefore, for case-control analyses of SCA and ASD, logistic regression analyses were weighted by a sex-normalization coefficient calculated for males and females in each control cohort by dividing half of the total number of samples in each cohort by the number of males or females in that cohort, respectively (Table S1). A newborn cohort was included in this study as a reference for SCA prevalence among newborns^19^. Logistic regression analyses using SPARK siblings and the newborn reference cohort as controls were also weighted, despite minimal sex biases. Cases were unweighted because the sex ratio of the ASD cases were male skewed (3.6:1) and matched the expected sex difference in ASD prevalence. SPARK probands are primarily ascertained through their parents, and therefore the sex ratio of the probands is not biased by the same selective pressures as population cohorts (the sex ratio of siblings of SPARK probands is nearly 1:1).

In order to compare results from the SPARKMC-SCA analyses with the results of the iPSYCH-SCA study^10^, we first calculated ORs for each association using counts of cases and controls in the iPSYCH-SCA cohort using logistic regression, weighting controls by the sex-normalization coefficient. ORs could not be calculated for comparisons including 45,X because the number of 45,X among ASD-negative controls in the iPSYCH-SCA cohort were not reported due to sample size. The results using the SPARKMC-SCA cohort were then meta-analyzed with data derived from the iPSYCH-SCA cohort using a fixed-effects model in metafor^43^. Two measures of heterogeneity, i^2^ and Q, were reported. The ORs for the extra X and Y effects were compared by calculating the z-statistic of the difference between the two ORs and compared to a normal distribution.

In the primary analyses, 848 females (0.90%) and 1,311 males (2.16%) with an ASD or ID diagnosis were removed from the MyCode cohort to generate the original ASD-negative control cohort. However, because MyCode is primarily an adult cohort, the ASD diagnosis rate may be lower than a pediatric cohort such as SPARK due to changes in ASD diagnosis rates over time^44^. Leaving some undiagnosed ASD cases in the control cohorts may bias the resulting associations between ASD and sex chromosome complement. To define a range of plausible prevalence of undiagnosed ASD in the control population, we simulated MyCode control populations under 1) different prevalence values of undiagnosed ASD and 2) fold-enrichment of sex chromosome aneuploidies among undiagnosed ASD cases. Since current estimates of ASD prevalence from the Center of Disease Control range from 1.14% in girls to 4.3% in boys^45^, we varied the simulated prevalence of undiagnosed ASD in males and females from 0 to 4% [e.g., the simulated prevalence of ASD among males in MyCode ranges from 2.16% (0% undiagnosed ASD) to 6.16% (4% undiagnosed ASD)]. Enrichment of each SCA group among ASD-negative controls was also varied from 0.5-fold (e.g., the SCA is protective against ASD) to 8-fold (e.g., the SCA is a strong risk factor for ASD). For each iteration of the parameters, a simulated control group was created by recalculating the number of those with and without SCAs after subtracting the estimated number of undiagnosed ASD cases. We then re-calculated the association between SCA and ASD for each simulated cohort.

Differences in dimensional measures, diagnosis age, and parental age between ASD-positive SPARK probands with and without SCA were calculated to assess whether SCAs were ascertained differently from euploids in SPARK. The associations between having any SCA and (1) SCQ, (2) VABS ABC score, (3) diagnosis age, (4) maternal age, and (5) paternal age, relative to controls were calculated using linear regression. Differences in performance for all cognitive tests among UKB participants were calculated using linear regression. Differences in educational attainment and household income among UKB and All of Us participants were calculated using ordered logistic regression.

To assess SCA ascertainment bias in population cohorts, the chromosomal sex-matched prevalence of each SCA was compared between each population cohort (MyCode, All of Us, UKB) and the newborn reference cohort using logistic regression. To examine the effect of genetic ancestry on our results, we repeated the analyses in a subset of participants with European ancestry. The European ancestry group was selected for sensitivity analyses as it is the largest subset in the SPARKMC-SCA cohort. The European ancestry subsets in the SPARKMC-SCA, UK Biobank, and All of Us cohorts were identified through principal component analysis and clustering with the 1000 Genomes reference population as performed in the UK Biobank^37^.

### Reporting summary

Further information on research design is available in the Nature Portfolio Reporting Summary linked to this article.

## Supplementary information

Supplementary Information

Peer Review File

Reporting Summary

## Source data

Source Data

## Data Availability

All analyses reported in this manuscript were performed on existing genomic and phenotypic datasets. Researchers can request access to the SPARK genetic and phenotypic data through by SFARI Base at: https://base.sfari.org. Researchers can register to access the UK Biobank resource at: https://www.ukbiobank.ac.uk. All sequencing data used in this study are available on the All of Us Researcher Workbench in the v7 release. Researchers can register to access this resource at: https://www.researchallofus.org/. The MyCode SCA dataset and scripts used in analyses can be made available by contacting the investigators directly. Source data are provided with this paper.

## Code availability

All code used for data preparation and analysis are available upon request by contacting Matthew Oetjens at mtoetjens@geisinger.edu.
