# Supplementary material for: A genome-first study of sex chromosome aneuploidies provides evidence of Y chromosome dosage effects on autism risk
Source: Nat Commun. 2024 Oct 15;15:8897. doi: 10.1038/s41467-024-53211-7 (PMC11480344; doi:10.1038/s41467-024-53211-7)
Supplement: Supplementary file 1 — Supplementary Information [file 41467_2024_53211_MOESM1_ESM.pdf]

Supplemental Methods

SCA Identification

Genotype data from both UK Biobank platforms were combined due to the consistency between them. For All of Us and the UK Biobank, mean LRRx and mean LRRy were used because the mean showed superior separation of sex chromosome copy number in those cohorts.

PAF, ARP, and Penetrance

Population attributable fraction (PAF) is the proportional reduction in a disorder that would occur if exposure to a risk factor were removed from the population. Attributable risk proportion (ARP) is an estimate of the proportion of risk among the exposed that can be directly attributed to the exposure of interest. PAF and ARP were calculated using the *twoxtwo* library in R[1], by multiplying the counts by the sex-matched normalization coefficient (Table S2). A Bayesian method that accounts for the oversampling of cases in our cohort relative to the general population, *CalPen*, was used to calculate the penetrance of ASD for each sex chromosome complement[2,3]. The latest sex-specific estimates of ASD prevalence among 8-year-old children (43.0 in 1,000 in boys and 11.4 in 1,000 girls) from the US Centers for Disease Control and Prevention (CDC) were used as baseline risk percentages[4].

Cognitive performance and related outcomes

To compare cognitive exam performance and social determinants of health by the sex chromosome complement, we used data collected on UK Biobank participants during

the baseline assessment. UK Biobank participants performed cognitive exams including Fluid Intelligence, Pairs Matching, Reaction Time, Digit Span, Symbol Digit, Numeric Trail Making, and Alphanumeric Trail Making. Educational attainment/qualifications and income were also available for UK Biobank participants as part of the assessment exam and via online follow-up. Results of cognitive tests were normalized following Kendall et al.[5] before converting into z-scores.

For the Fluid Intelligence test (fields 20016 and 20191), the total number of correct answers was used as the outcome. For the Pairs Matching test (field 399), the total number of errors was used as the outcome and a log + 1 transformation was applied, excluding those who did not finish the test (fewer than six correct matches (field 398)). For the Reaction Time test (field 20023), the mean reaction time to correct response was used as the outcome, and a log transformation was applied, excluding outliers (<500 milliseconds and >1500 milliseconds). For the Digit Span test (fields 4282 and 20240), the maximum number of digits remembered was used as the outcome. For the Symbol Digit Substitution test (fields 23324 and 20159), the number of correct substitutions was used as the outcome, excluding outliers (<3 and >36 substitutions). For the Numeric Trail Making (fields 6348 and 20156) and Alphanumeric Trail Making (fields 6350 and 20157) tests, time taken to complete the tests was used as the outcome, and a log transformation was applied, excluding times of zero.

For all analyses of cognitive performance, only data from UK Biobank participants who completed the entire test were used. For each of the five cognitive tests that were available both in-person and online, the online score was only used if the participant did not complete it in-person.

Annual household income in the UK Biobank was grouped into five ordinal brackets based on responses to the baseline assessment field 738 “Average total household income before tax” (<£18K, £18K to £30,999, £31K to £51,999, £52 to £100K, >£100K). Individuals who responded “Do not know”, “Prefer not to answer”, or who didn’t respond were excluded. Educational attainment in the UK Biobank was grouped into six ordinal brackets indicating an individual’s highest qualification obtained based on responses to the baseline assessment field 6138 “Qualifications” (no additional qualifications, NVQ or HND or HNC or equivalent, CSEs or equivalent, O levels/GCSEs or equivalent, A levels/AS levels or equivalent, and College or University degree). Individuals who responded “Other professional qualifications”, “Prefer not to answer”, or who didn’t respond were excluded. Annual household income in All of Us was grouped into nine ordinal brackets based on responses to the survey question “Income: Annual Income” (<\$10K, \$10K to \$25K, \$25K to \$35K, \$35K to \$50K, \$50K to \$75K, \$75K to \$100K, \$100K to \$150K, \$150K to \$200K, >\$200K). Educational attainment in All of Us was grouped into eight ordinal brackets indicating an individual’s highest grade completed based on responses to the survey question “Education Level: Highest Grade” (none, 1st, 5th, 9th, 12th or GED, some college, college graduate, advanced degree).

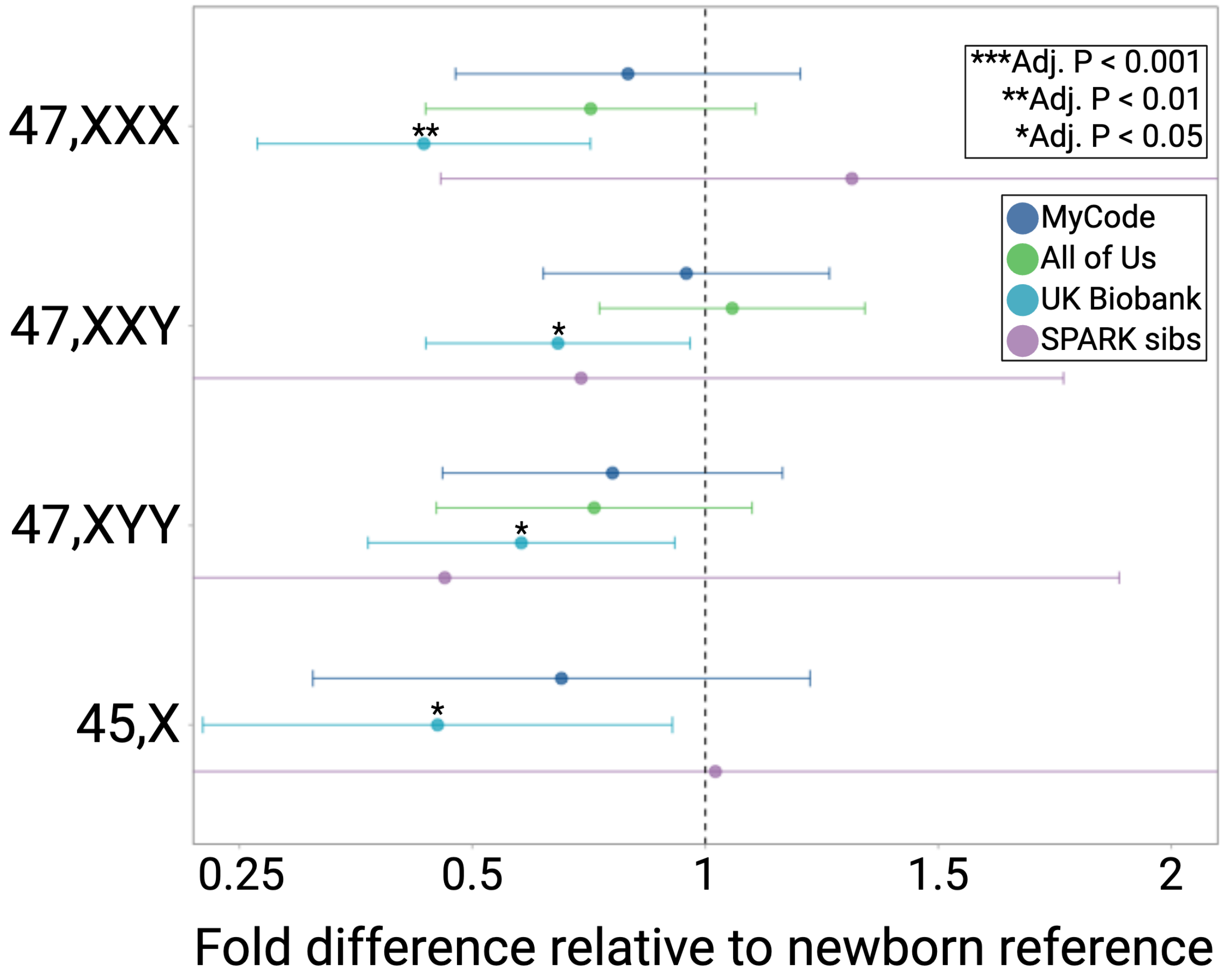

Figure S1. SCA prevalence in control cohorts relative to newborn reference cohort. Forest plots show the prevalence of each SCA relative to the newborn reference cohort (n=34,904). Points represent coefficients calculated using generalized linear models, and error bars represent the associated 95% confidence intervals for MyCode (n=152,331), All of Us (n=308,248), the UK Biobank (n=487,865), and ASD-negative SPARK siblings (n=3,683). Asterisks denote level of statistical significance for each 2-sided test, adjusted for multiple testing using Benjamini-Hochberg false discovery rate correction[6]. Source data are provided as a Source Data file.

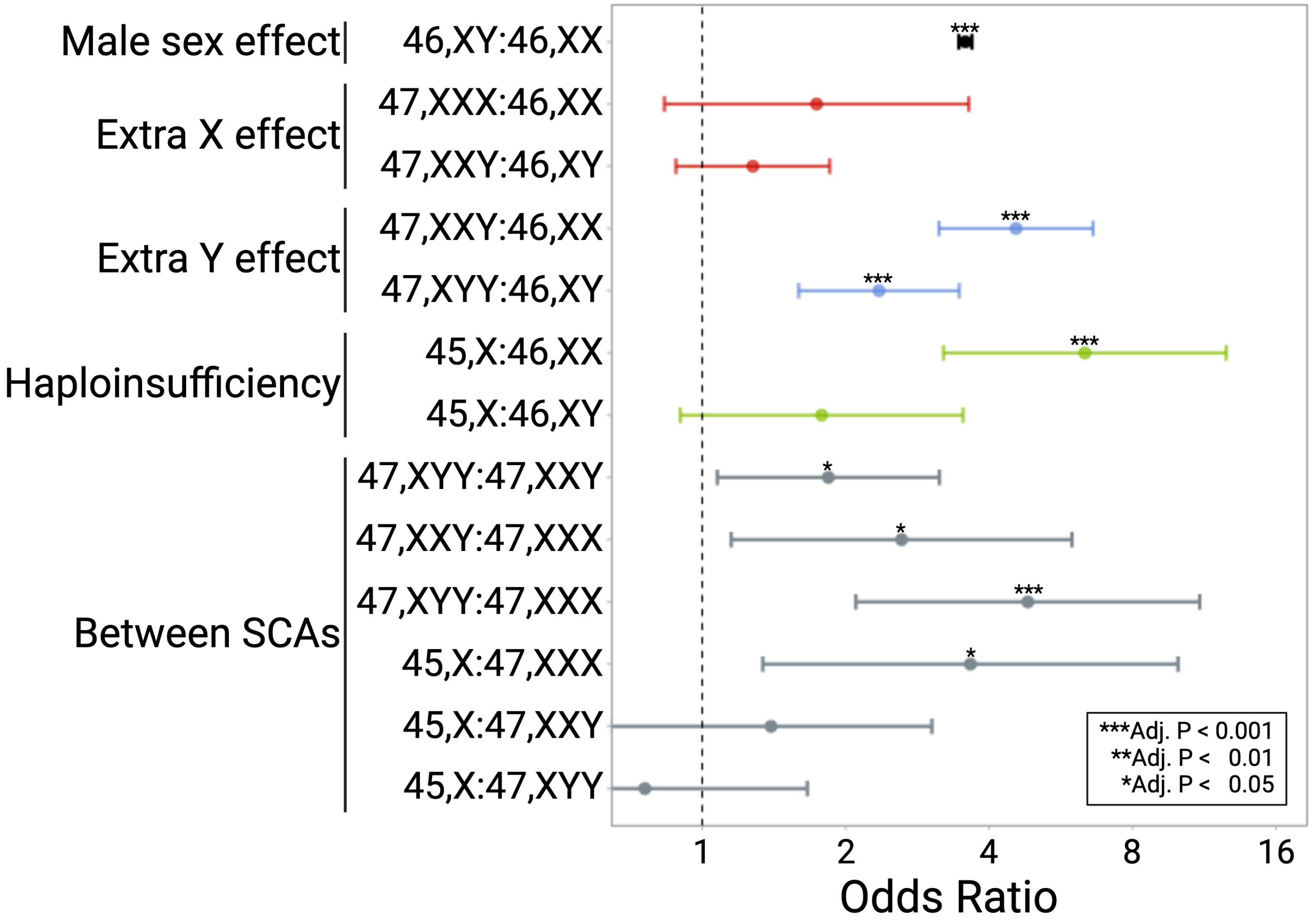

Figure S2. Risk of autism by sex chromosome complement including all between-SCA comparisons. Forest plot shows the results of logistic regression for each comparison. Points denote calculated odds ratio and error bars represent 95% confidence intervals. The groupings on the Y-axis and colors indicate the central hypothesis from Green et al. tested in the comparison. Asterisks denote level of statistical significance for each 2-sided test, adjusted for multiple testing using Benjamini-Hochberg false discovery rate correction. Sample sizes used to derive statistics for each sex chromosome complement are as follows: 46,XX, 98,277; 46,XY, 78,790; 47,XXY, 125; 47,XYY, 94; 47,XXX, 86; 45,X, 44. Source data are provided as a Source Data file.

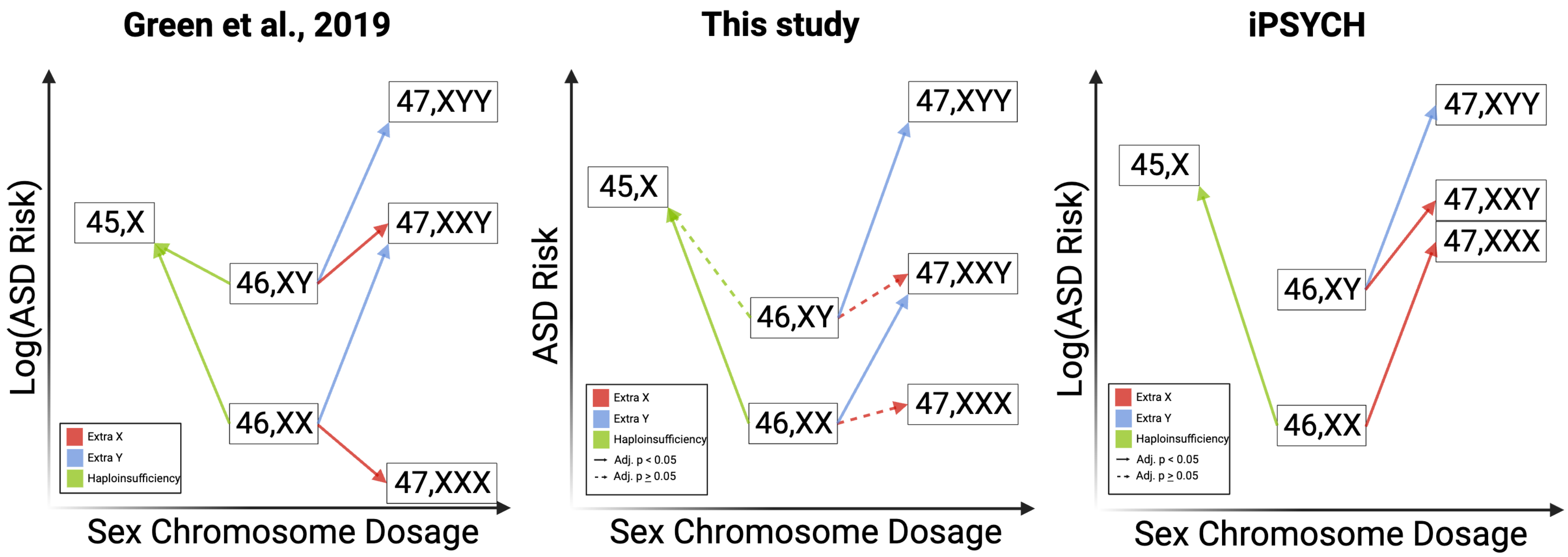

Figure S3. Comparison of the Green et al. 2019 model across two large ASD case-control cohorts. Plots show the effects of sex chromosome dosage on autism risk in those with a sex chromosome aneuploidy relative to those with two sex chromosomes. A) Plot summarizes the Green et al., 2019 model which is based on the autism prevalence reported by clinical studies of sex chromosome aneuploidies. B) Plot summarizes the results of analyses performed here in the SPARKMC-SCA cohort. C) Plot summarizes the results of analyses performed by Sánchez et al., 2023 in the Danish iPSYCH-SCA study. Solid lines in panels B and C indicate the association is statistically significant at adjusted P < 0.05 while dashed lines indicate adjusted P ≥ 0.05. P-values were not reported in the Green et al., 2019 model. Autism risk in panels A and C are log-transformed to emphasize the consistent patterns between studies.

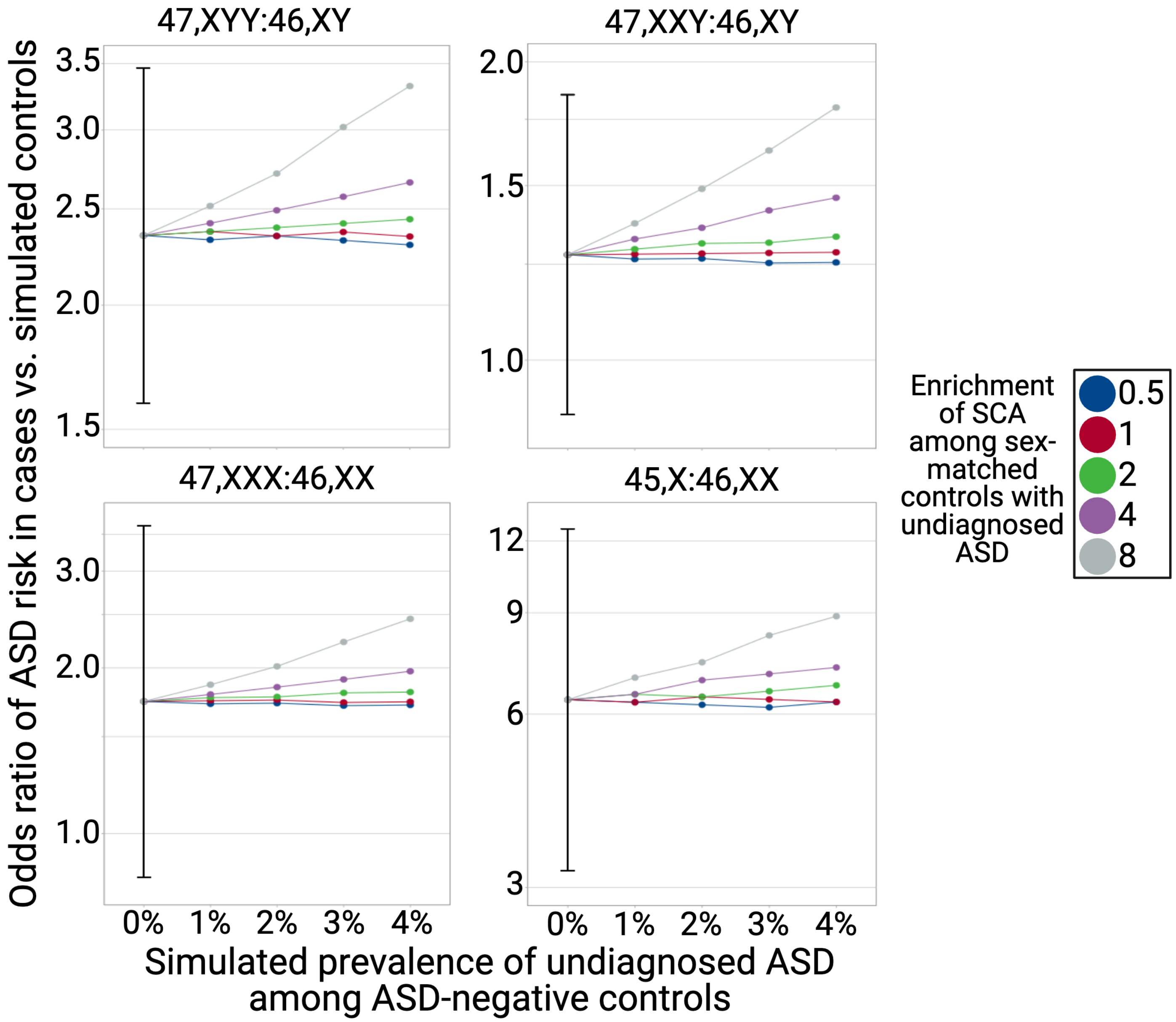

Figure S4. Simulating undiagnosed ASD-negative controls in the SPARKMC-SCA cohort. ASD may be underdiagnosed among the ASD-negative control population relative to the ASD-positive case population. 848 females (0.90%) and 1,311 males (2.16%) with ASD or ID were removed from the MyCode cohort to generate the original ASD-negative control cohort. In order to determine the effect of undiagnosed ASD among the ASD-negative control population on the resulting odds ratio of ASD risk, simulated control populations were generated by varying 1) the prevalence of undiagnosed ASD among ASD-negative controls without SCA, and 2) the enrichment of SCA among sex-matched controls with undiagnosed ASD. The simulated prevalences of undiagnosed ASD among ASD-negative controls on the x-axis range from 0%, meaning that ASD was not underdiagnosed in the control population and the control (e.g. the original calculation from Figure 2), to 4%, meaning that an additional 4% of

individuals with 46,XY or 46,XX were removed from the control population, along with a proportional number of individuals with an SCA depending on the enrichment of SCA among sex-matched controls with undiagnosed ASD. 0.5 enrichment of SCA (blue) simulates a population where the prevalence of SCA is lower than the prevalence of euploids among those with undiagnosed ASD; 1.0 enrichment of SCA (red) simulates a population where the prevalence of SCA and euploids among undiagnosed ASD are the same; and enrichment >1 simulates a population where SCAs are 2-, 4-, and 8-fold more prevalent than euploids among undiagnosed ASD cases (green, purple, and gray, respectively). Each point denotes the odds ratio calculated from logistic regression for each comparison. The originally calculated odds ratios and corresponding 95% confidence intervals are shown for each comparison at the 0% line. No simulated odds ratios exceed the bounds of the original 95% confidence interval. Sample sizes used to derive statistics for each sex chromosome complement are as follows: 46,XX, 98,277; 46,XY, 78,790; 47,XXY, 125; 47,XYY, 94; 47,XXX, 86; 45,X, 44. Source data are provided as a Source Data file.

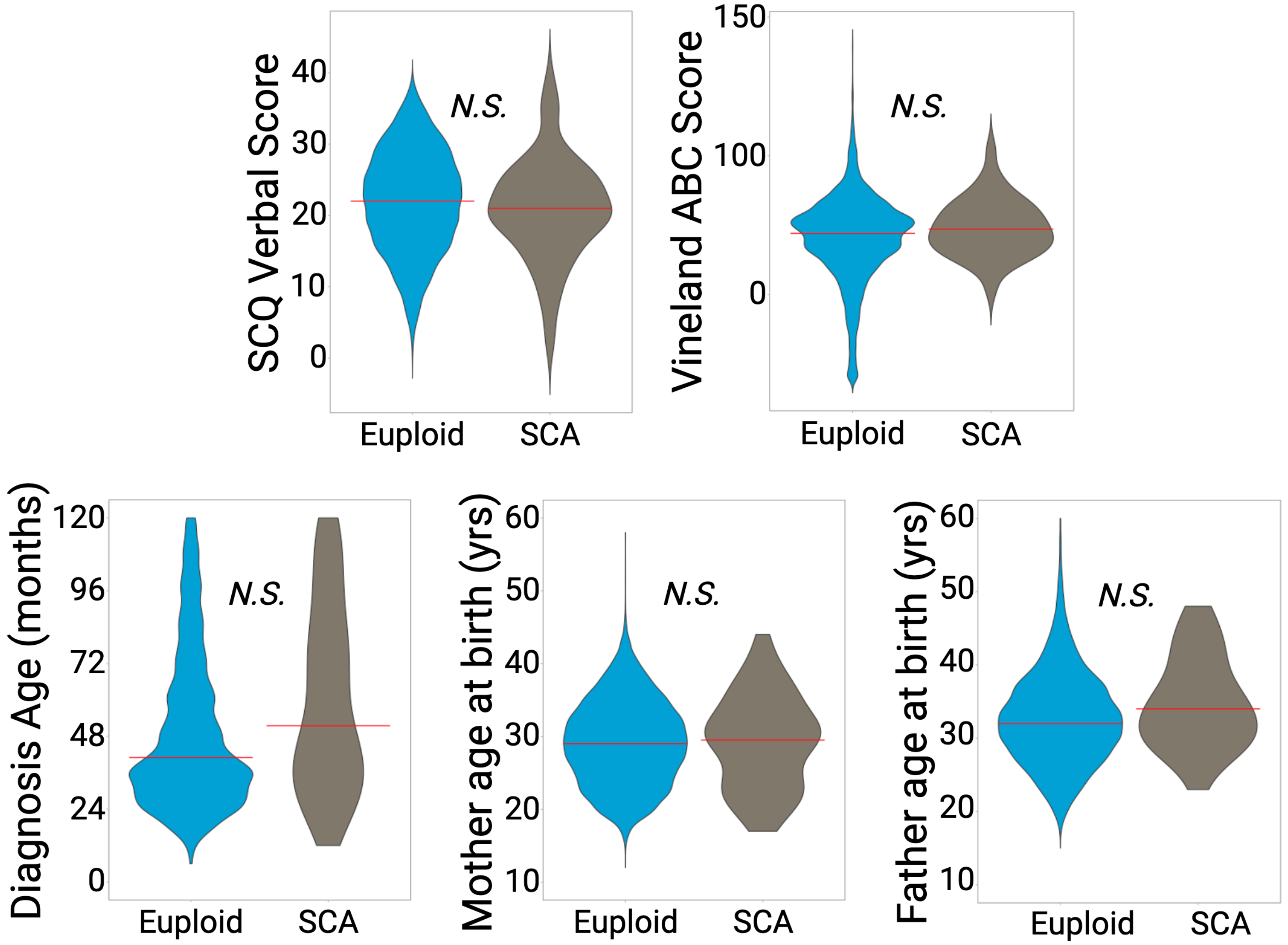

Figure S5. Ascertainment metrics between SPARK participants with a sex chromosome aneuploidy (SCA) and those without (Euploid). Participants with and without an SCA were compared across five metrics: Social Communication Questionnaire (SCQ) final score for verbal participants only (n=14,644), Vineland Adaptive Behavior Scales (VABS) ABC score (n=7,275), Diagnosis age (in months) (n=25,080), Maternal age at proband birth (in years) (n=14,418), and paternal age at proband birth (in years) (n=8,103). For each metric, a 2-sided Fisher's exact test was performed to determine whether the groups were significantly different. No metrics were significantly different between groups (P > 0.05 for all comparisons). Source data are provided as a Source Data file.

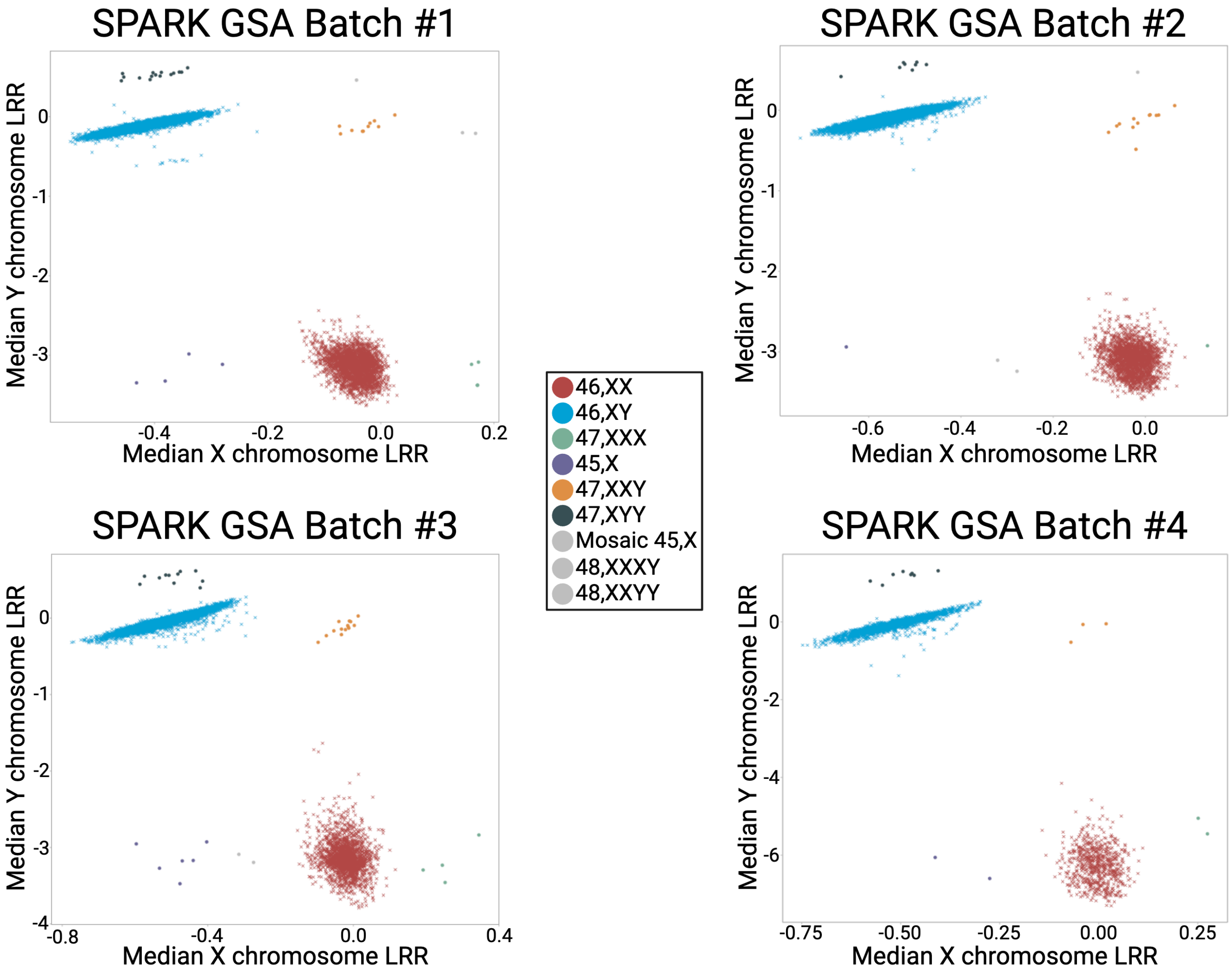

Figure S6. Identifying SCAs in SPARK. Log R Ratio (LRR) plots are shown for each batch from the SPARK cohort. SCA were identified using the median Y chromosome LRR and median X chromosome LRR. Each identified SCA was then visually confirmed. Participants with mosaic 45,X; 48,XXXY; and 48,XXYY are shown in gray and were not used in analyses. Source data are provided as a Source Data file.

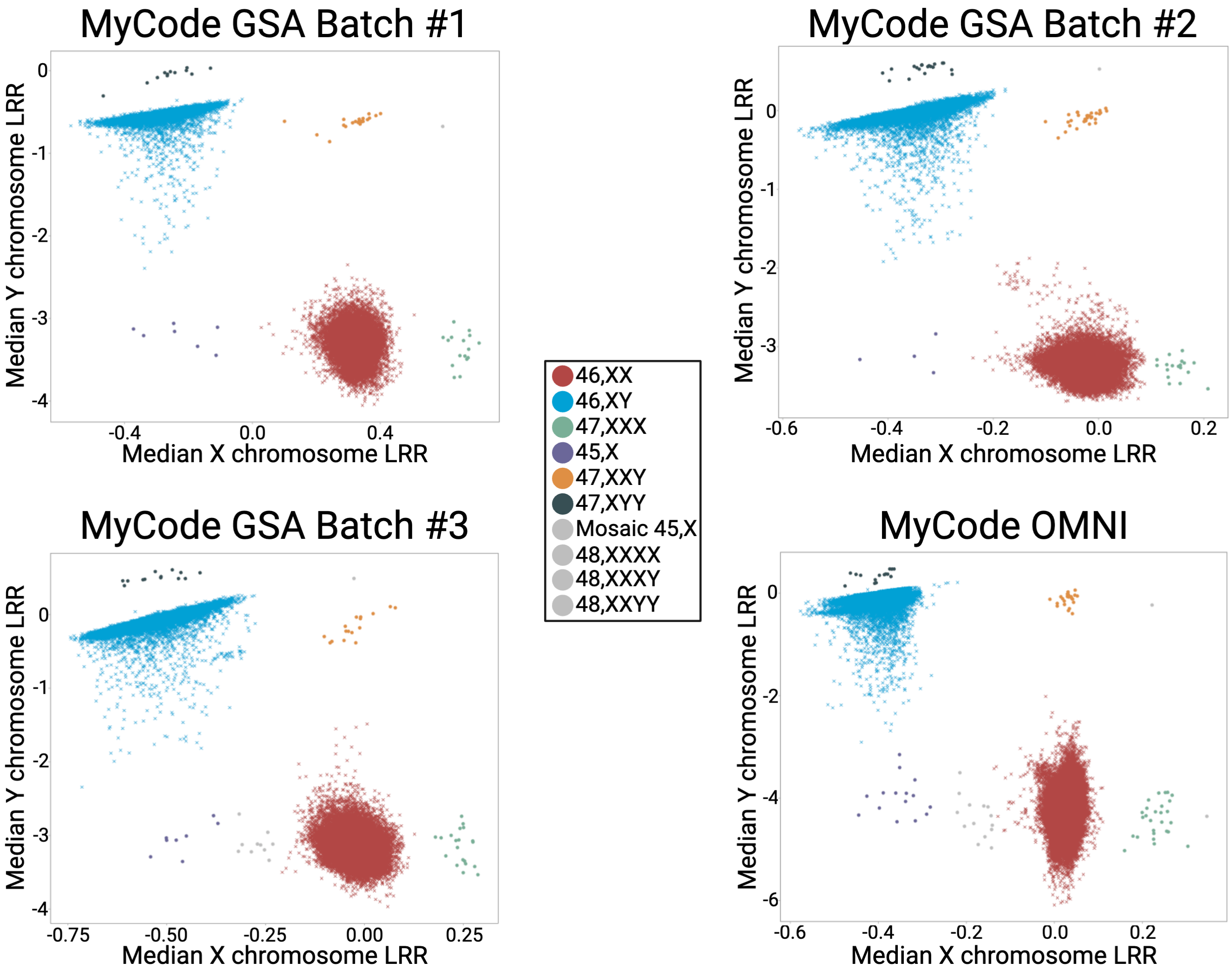

Figure S7. Identifying SCAs in MyCode. Log R Ratio (LRR) plots are shown for each batch from the MyCode cohort. SCA were identified using the median Y chromosome LRR and median X chromosome LRR. Each identified SCA was then visually confirmed. Participants with mosaic 45,X; 48,XXXX; 48,XXXY; and 48,XXYY are shown in gray and were not used in analyses. Source data are provided as a Source Data file.

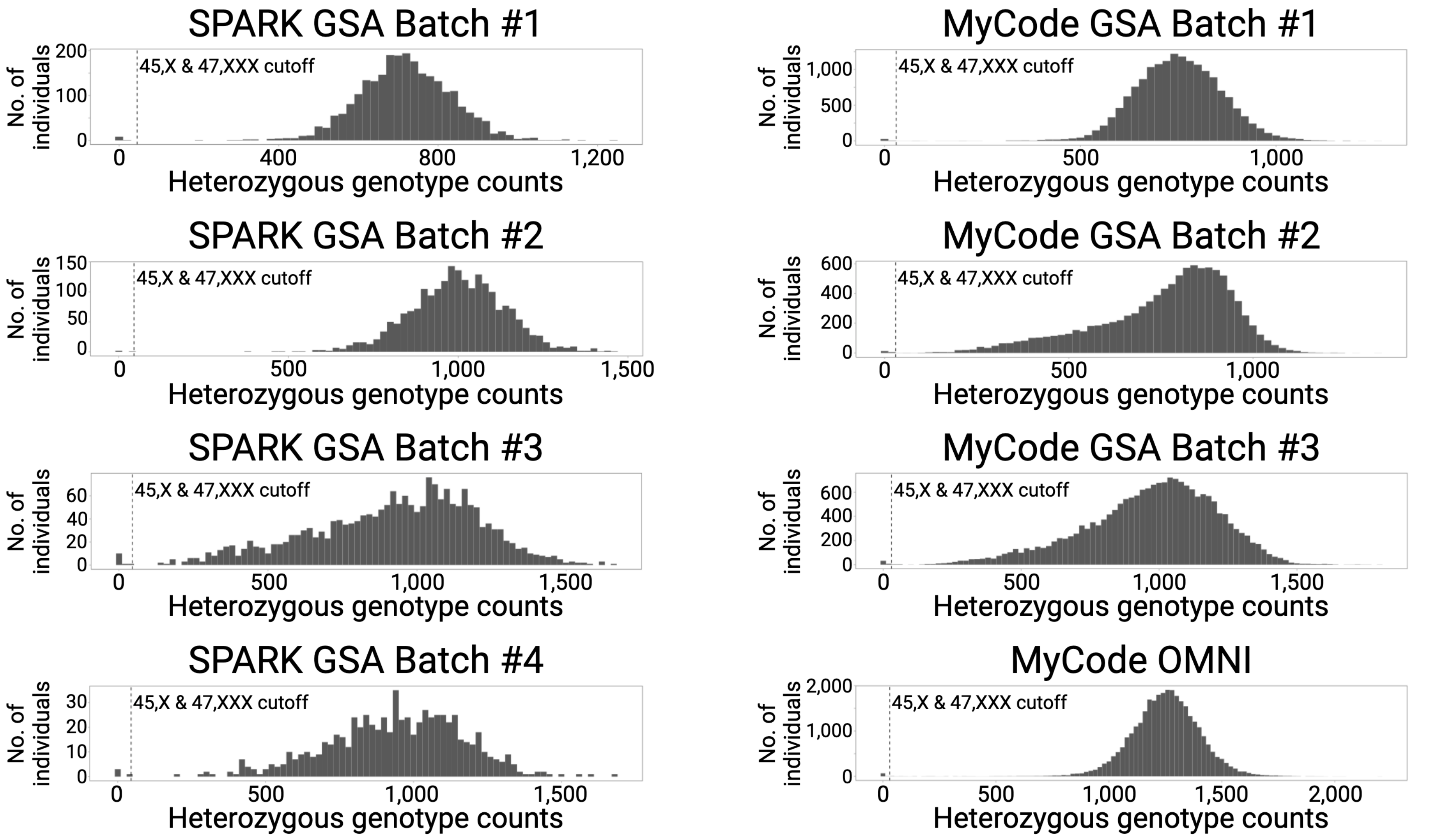

Figure S8. Heterozygous genotype counts across the X chromosome. The number of heterozygous markers across the X chromosome are shown for female participants in each batch from the SPARK and MyCode cohorts. Bars in each histogram have a width of twenty markers. Only participants with heterozygous genotype counts below the threshold indicated by the dashed line could be considered 45,X or 47,XXX. Source data are provided as a Source Data file.

Table S1. Case and control population descriptions.

	SPARK probands	MyCode	UK Biobank	All of Us	Newborn reference	SPARK siblings	iPSYCH-SCA cases	iPSYCH-SCA controls
**Case/control**	Case	Control	Control	Control	Control	Control	Case	Control
**ASD & ID +/-**	ASD +	ASD - & ID -	ASD - & ID -	ASD - & ID -	NA[1]	ASD -	ASD +	NA[1]
**Region of origin**	United States	United States	United Kingdom	United States	Denmark	United States	Denmark	
**Age, mean (SD), years**	11.5 (8.9)	56.8 (18.1)	56.5 (8.1)	55.6 (17.0)	NA (all newborn)	7.6 (4.6)	10.9 (3.5)[2]	
**Males**	19,590	59,419	223,452	121,950	17,868	1,846	16,768	21,522
**Females**	5,495	92,912	264,413	186,298	17,036	1,837	5,432	21,132
**Total**	25,085	152,331	487,865	308,248	34,904	3,683	22,200	42,654
**Male normalization coefficient**	1	1.281838	1.091655	1.2638294	0.976718	0.997562	1	0.99094
**Female normalization coefficient**	1	0.81976	0.922544	0.8272982	1.024419	1.00245	1	1.009228

Race/Ethnicity[3], %								
**White**	71.5	94.8	94.2	51.7	NA	16.8	NA	NA
**African**	9.4	1.9	0.8	20.6	NA	0.6	NA	NA
**Asian**	3.3	0.4	2.4	3.2	NA	0.9	NA	NA
**Latino**	9.5	2.5	0	1.9	NA	4.5	NA	NA
**Other**	3	0.2	2.1	1.9	NA	0.3	NA	NA
**Unknown**	3.3	0.2	0.5	20.8	NA	76.9	NA	NA

[1]Individuals were chosen at random from the population without regard for ASD or ID status.
[2]Mean (SD) age was reported for the entire iPSYCH cohort. Ages for ASD cases and controls were not reported separately.
[3]Race/Ethnicity in SPARK, UK Biobank, and All of Us is self-reported. Race in MyCode is as documented in the electronic health record.
ASD=autism spectrum disorder; ID=intellectual disability

Table S2. SCA counts and prevalence by cohort.

	47,XXY	47,XYY	47,XXX	45,X
**SPARK, counts (sex-matched prevalence per 100K)**	37 (189)	41 (209)	8 (146)	12 (218)
**MyCode, counts (sex-matched prevalence per 100K)**	88 (148)	53 (89)	78 (84)	32 (34)
**UK Biobank, counts (sex-matched prevalence per 100K)**	226 (101)	152 (68)	121 (46)	63 (24)
**All of Us, counts (sex-matched prevalence per 100K)**	207 (170)	103 (84)	140 (75)	NA
**SPARK siblings, counts (sex-matched prevalence per 100K)**	2 (108)	1 (54)	3 (163)	1 (54)
**Newborn reference, counts (sex-matched prevalence per 100K)**	28 (157)	21 (118)	18 (106)	9 (53)
**iPSYCH ASD + cases, counts (sex-matched prevalence per 100K)**	60 (358)	73 (435)	14 (258)	8 (147)
**iPSYCH ASD - controls, counts (sex-matched prevalence per 100K)**	27 (125)	16 (74)	14 (66)	4 (19)

Table S3. Associations between SCA and ASD in the SPARKMC-SCA cohort using a stricter 80% mosaicism threshold to define 45,X. Odds ratios and associated 95% confidence intervals (CI) represent the result of logistic regression. The p-value for each 2-sided test was adjusted using Benjamini-Hochberg false discovery rate correction (Adj. p).

Effect	Comparison	OR (95% CI)	Adjusted P
Haploinsufficiency	45,X:46,XX	6.8 (2.6-18)	3.05E-4
Haploinsufficiency	45,X:46,XY	1.9 (0.7-5)	1.98E-01

SCA=sex chromosome aneuploidy; ASD=autism spectrum disorder; OR=odds ratio

Table S4. Penetrance, PAF, and ARP among SCA with significantly elevated ASD risk in the SPARKMC-SCA cohort.

	Penetrance (%) (95% CI)	Population Attributable Fraction (%) (95% CI)	Attributable Risk Proportion rel. to 46,XX (%) (95% CI)	Attributable Risk Proportion rel. to 46,XY (%) (95% CI)
**46,XY**	*NA*	51.9 (50.8-52.9)	67.1 (66.2-68.1)	*NA*
**47,XXY**	5.5 (3.3-8.9)	0.063 (0.021-0.105)	72.8 (65.1-80.4)	*N.S.*
**47,XYY**	9.6 (5.6-15.8)	0.102 (0.058-0.146)	82.2 (77.8-86.5)	45.7 (32.5-58.8)
**45,X**	7.0 (2.9-15.3)	0.026 (0.003-0.050)	78.7 (68.8-88.7)	35.3 (5.0-65.6)
**47,XXX**	2.1 (0.8-4.7)	*NA*	*N.S.*	*N.S.*

SCA=sex chromosome aneuploidy; ASD=autism spectrum disorder

Table S5. Association between SCA and ASD in the SPARKMC-SCA and iPSYCH-SCA cohorts. Odds ratios and associated 95% confidence intervals (CI) and standard errors (SE) represent the result of logistic regression. The p-value for each 2-sided test was adjusted using Benjamini-Hochberg false discovery rate correction (Adj. p).

Comparison	Cohort	OR (95% CI)	SE	Adjusted P	HR (95% CI)*
47,XXX:46,XX	SPARKMC-SCA	1.7 (0.8-3.6)	0.38	1.49E-01	NA
47,XXX:46,XX	iPSYCH-SCA	3.9 (1.9-8.2)	0.38	5.98E-04	4.7 (2.1-10.3)
47,XXY:46,XY	SPARKMC-SCA	1.3 (0.9-1.9)	0.19	1.97E-01	NA
47,XXY:46,XY	iPSYCH-SCA	2.9 (1.8-4.5)	0.23	1.77E-05	2.7 (1.7-4.3)
47,XYY:46,XY	SPARKMC-SCA	2.4 (1.6-3.5)	0.20	4.06E-05	NA
47,XYY:46,XY	iPSYCH-SCA	5.9 (3.4-10.1)	0.28	5.71E-10	5.6 (3.4-9.4)
47,XXY:46,XX	SPARKMC-SCA	4.6 (3.1-6.6)	0.19	8.46E-15	NA
47,XXY:46,XX	iPSYCH-SCA	8.8 (5.6-13.9)	0.23	7.81E-20	NA
47,XYY:47,XXY	SPARKMC-SCA	1.8 (1.1-3.1)	0.27	3.34E-02	NA
47,XYY:47,XXY	iPSYCH-SCA	2.1 (1-4.2)	0.36	5.60E-02	NA
47,XXY:47,XXX	SPARKMC-SCA	2.6 (1.2-6)	0.42	3.02E-02	NA
47,XXY:47,XXX	iPSYCH-SCA	2.3 (0.9-5.4)	0.44	7.34E-02	NA

*HRs were calculated for the iPSYCH cohort by Sanchez et al., 2023 for three of the comparisons shown here.
SCA=sex chromosome aneuploidy; ASD=autism spectrum disorder; OR=odds ratio; CI=confidence interval; SE=standard error; HR=hazard ratio

Table S6. Meta-analysis of the results from the SPARKMC-SCA and iPSYCH-SCA cohorts. Odds ratios and associated 95% confidence intervals, standard errors, I^2, Q, and Q p-value, were calculated for each meta-analysis using a fixed-effects model in metafor[7]. P-values for each 2-sided test were adjusted using Benjamini-Hochberg false discovery rate correction (Adj. p).

Comparison	OR (95% CI)	SE	Adjusted P	I^2	Q	Q p-value
47,XXX:46,XX	2.6 (1.5 - 4.4)	0.266	5.98E-04	56.684	2.309	0.129
47,XXY:46,XY	1.8 (1.3 - 2.4)	0.147	2.53E-04	86.242	7.269	0.007
47,XYY:46,XY	3.2 (2.3 - 4.4)	0.161	2.16E-12	86.260	7.278	0.007
47,XXY:46,XX	5.9 (4.4 - 7.9)	0.147	1.96E-32	79.360	4.845	0.028
47,XYY:47,XXY	1.9 (1.2 - 2.9)	0.218	4.79E-03	0.000	0.058	0.809
47,XXY:47,XXX	2.4 (1.3 - 4.4)	0.305	5.03E-03	0.000	0.058	0.810

SCA=sex chromosome aneuploidy; ASD=autism spectrum disorder; OR=odds ratio; CI=confidence interval; SE=standard error

Table S7. Associations between SCA and ASD in the SPARKMC-SCA cohort when not removing individuals with ID in the control group. Odds ratios and associated 95% confidence intervals (CI) and standard errors (SE) represent the result of logistic regression. The p-value for each 2-sided test was adjusted using Benjamini-Hochberg false discovery rate correction (Adj. p).

Effect	Comparison	OR (95% CI)	SE	Adjusted P
Male sex effect	46,XY:46,XX	3.6 (3.5 - 3.7)	0.016	0.00E+00
Extra X effect	47,XXX:46,XX	1.7 (0.8 - 3.5)	0.374	1.84E-01
Extra X effect	47,XXY:46,XY	1.3 (0.9 - 1.8)	0.189	1.98E-01
Extra Y effect	47,XXY:46,XX	4.6 (3.1 - 6.6)	0.190	6.21E-15
Extra Y effect	47,XYY:46,XY	2.2 (1.5 - 3.2)	0.195	1.58E-04
Haploinsufficiency	45,X:46,XX	6.2 (3.1 - 12.2)	0.347	4.34E-07
Haploinsufficiency	45,X:46,XY	1.7 (0.9 - 3.4)	0.347	1.42E-01
Between SCAs	47,XXY:47,XXX	2.7 (1.2 - 6.1)	0.419	3.20E-02
Between SCAs	47,XYY:47,XXY	1.7 (1 - 2.9)	0.271	7.58E-02

SCA=sex chromosome aneuploidy; ASD=autism spectrum disorder; ID=intellectual disabilities; OR=odds ratio; CI=confidence interval; SE=standard error

Table S8. Associations between SCA and ASD across cohorts. Odds ratios and associated 95% confidence intervals (CI) represent the result of logistic regression. The p-value for each 2-sided test was adjusted using Benjamini-Hochberg false discovery rate correction (Adj. p).

Effect	Comparison	Control Cohort	OR (95% CI)	Unadjusted P	Adjusted P
Male sex effect	46,XY:46,XX	All of Us	3.6 (3.5-3.7)	0.00E+00	0.00E+00
Male sex effect	46,XY:46,XX	MyCode	3.6 (3.5-3.7)	0.00E+00	0.00E+00
Male sex effect	46,XY:46,XX	Nielsen	3.6 (3.4-3.7)	0.00E+00	0.00E+00
Male sex effect	46,XY:46,XX	SPARK sibs	3.6 (3.3-3.8)	2.08E-267	2.08E-266
Male sex effect	46,XY:46,XX	UKB	3.6 (3.5-3.7)	0.00E+00	0.00E+00
Extra X effect	47,XXX:46,XX	All of Us	1.9 (0.9-4)	6.94E-02	1.11E-01
Extra X effect	47,XXX:46,XX	MyCode	1.7 (0.8-3.6)	1.41E-01	2.01E-01
Extra X effect	47,XXX:46,XX	Nielsen	1.4 (0.6-3.2)	4.46E-01	5.19E-01
Extra X effect	47,XXX:46,XX	SPARK sibs	0.9 (0.2-3.4)	8.67E-01	8.85E-01
Extra X effect	47,XXX:46,XX	UKB	3.2 (1.6-6.5)	1.54E-03	4.04E-03
Extra X effect	47,XXY:46,XY	All of Us	1.1 (0.8-1.6)	5.38E-01	5.72E-01
Extra X effect	47,XXY:46,XY	MyCode	1.3 (0.9-1.9)	1.97E-01	2.59E-01
Extra X effect	47,XXY:46,XY	Nielsen	1.2 (0.7-2)	4.56E-01	5.19E-01
Extra X effect	47,XXY:46,XY	SPARK sibs	1.7 (0.4-7.3)	4.43E-01	5.19E-01
Extra X effect	47,XXY:46,XY	UKB	1.9 (1.3-2.6)	3.82E-04	1.19E-03
Extra Y effect	47,XXY:46,XX	All of Us	4 (2.8-5.6)	4.58E-15	2.86E-14
Extra Y effect	47,XXY:46,XX	MyCode	4.6 (3.1-6.6)	1.41E-15	1.01E-14
Extra Y effect	47,XXY:46,XX	Nielsen	4.3 (2.6-7.1)	7.54E-09	3.43E-08
Extra Y effect	47,XXY:46,XX	SPARK sibs	6.2 (1.5-25.9)	1.19E-02	2.71E-02
Extra Y effect	47,XXY:46,XX	UKB	6.7 (4.7-9.4)	6.77E-27	5.64E-26
Extra Y effect	47,XYY:46,XY	All of Us	2.5 (1.7-3.5)	3.97E-07	1.53E-06
Extra Y effect	47,XYY:46,XY	MyCode	2.4 (1.6-3.5)	1.58E-05	5.64E-05
Extra Y effect	47,XYY:46,XY	Nielsen	1.8 (1-3)	3.26E-02	6.03E-02
Extra Y effect	47,XYY:46,XY	SPARK sibs	3.9 (0.5-28)	1.80E-01	2.44E-01
Extra Y effect	47,XYY:46,XY	UKB	3.1 (2.2-4.3)	1.11E-10	5.54E-10
Haploinsufficiency	45,X:46,XX	MyCode	6.4 (3.2-12.6)	1.14E-07	4.76E-07
Haploinsufficiency	45,X:46,XX	Nielsen	4.1 (1.8-9.8)	1.18E-03	3.48E-03
Haploinsufficiency	45,X:46,XX	SPARK sibs	4 (0.5-30.8)	1.81E-01	2.44E-01
Haploinsufficiency	45,X:46,XX	UKB	9.2 (4.9-17.1)	2.75E-12	1.53E-11
Haploinsufficiency	45,X:46,XY	MyCode	1.8 (0.9-3.5)	9.76E-02	1.44E-01
Haploinsufficiency	45,X:46,XY	Nielsen	1.2 (0.5-2.7)	7.33E-01	7.64E-01
Haploinsufficiency	45,X:46,XY	SPARK sibs	1.1 (0.1-8.7)	9.08E-01	9.08E-01
Haploinsufficiency	45,X:46,XY	UKB	2.6 (1.4-4.8)	2.84E-03	7.10E-03
Between SCAs	45,X:47,XXX	MyCode	3.7 (1.3-10)	1.13E-02	2.70E-02
Between SCAs	45,X:47,XXX	Nielsen	3 (0.9-9.9)	7.13E-02	1.11E-01
Between SCAs	45,X:47,XXX	SPARK sibs	4.5 (0.4-51.2)	2.25E-01	2.89E-01

Between SCAs	45,X:47,XXX	UKB	2.9 (1.1-7.4)	2.89E-02	5.55E-02
Between SCAs	47,XXY:47,XXX	All of Us	1.4 (0.6-3)	4.02E-01	4.90E-01
Between SCAs	47,XXY:47,XXX	MyCode	0.8 (0.3-1.7)	4.89E-01	5.44E-01
Between SCAs	47,XXY:47,XXX	Nielsen	2 (0.9-4.5)	7.72E-02	1.17E-01
Between SCAs	47,XXY:47,XXX	SPARK sibs	2.6 (1.2-6)	2.18E-02	4.54E-02
Between SCAs	47,XXY:47,XXX	UKB	3.1 (1.2-8.2)	2.10E-02	4.54E-02
Between SCAs	47,XYY:47,XXY	All of Us	7 (1-48.8)	5.05E-02	8.70E-02
Between SCAs	47,XYY:47,XXY	MyCode	2.1 (0.9-4.6)	6.91E-02	1.11E-01
Between SCAs	47,XYY:47,XXY	Nielsen	4.8 (2.1-11.1)	2.06E-04	6.87E-04
Between SCAs	47,XYY:47,XXY	SPARK sibs	2.2 (1.4-3.6)	1.41E-03	3.93E-03
Between SCAs	47,XYY:47,XXY	UKB	1.8 (1.1-3.1)	2.60E-02	5.20E-02

SCA=sex chromosome aneuploidy; ASD=autism spectrum disorder; OR=odds ratio; CI=confidence interval

Table S9. Demographics and SCA counts of European subsets

	**SPARK ASD+ Cases**	**MyCode ASD– Controls**	**UK Biobank ASD– Controls**	**All of Us ASD– Controls**
**Total counts**	16,843	143,277	460,451	168,348
**Age, mean (SD), years**	12.4 (9.5)	57.6 (17.9)	56.8 (8.0)	59.5 (17.0)
**Sex, % Female**	22.7	60.7	54.2	59.6
**SCA, counts (%)**				
47,XXY	26 (0.15)	87 (0.06)	216 (0.05)	94 (0.06)
47,XYY	25 (0.15)	50 (0.03)	143 (0.03)	50 (0.03)
47,XXX	6 (0.04)	78 (0.05)	115 (0.02)	59 (0.04)
45,X	12 (0.07)	32 (0.02)	61 (0.01)	*NA*

SCA=sex chromosome aneuploidy; ASD=autism spectrum disorder; SD=standard deviation

Table S10. Association between SCA and ASD in the European ancestry subset of the SPARKMC-SCA cohort. Odds ratios and associated 95% confidence intervals (CI) represent the result of logistic regression. The p-value for each 2-sided test was adjusted using Benjamini-Hochberg false discovery rate correction (Adj. p).

Effect	Comparison	OR (95% CI)	Unadjusted P	Adjusted P
Male sex effect	46,XY:46,XX	3.4 (3.3-3.5)	0.00E+00	0.00E+00
Extra X effect	47,XXX:46,XX	1.8 (0.8-4.1)	1.88E-01	2.09E-01
Extra X effect	47,XXY:46,XY	1.3 (0.8-2)	2.35E-01	2.35E-01
Extra Y effect	47,XXY:46,XX	4.4 (2.9-6.8)	1.06E-11	5.29E-11
Extra Y effect	47,XYY:46,XY	2.2 (1.4-3.4)	1.05E-03	2.63E-03
Haploinsufficiency	45,X:46,XX	8.6 (4.3-16.9)	7.42E-10	2.47E-09
Haploinsufficiency	45,X:46,XY	2.5 (1.3-5)	8.28E-03	1.38E-02
Between SCAs	45,X:47,XXX	4.9 (1.7-14.4)	4.03E-03	8.07E-03
Between SCAs	47,XXY:47,XXX	2.5 (1-6.4)	5.39E-02	7.70E-02
Between SCAs	47,XYY:47,XXY	1.7 (0.9-3.1)	1.09E-01	1.37E-01

SCA=sex chromosome aneuploidy; ASD=autism spectrum disorder; OR=odds ratio; CI=confidence interval

Table S11. Cognitive performance, income, and educational attainment measurements associated with SCA in European subset of the UK Biobank and All of Us cohorts. Coefficients and associated 95% confidence intervals (CI) and standard errors (SE) represent the result of linear regression analyses performed on the z-scored results of each of seven exams. Coefficients and associated 95% confidence intervals (CI) and standard errors (SE) for household income and educational attainment represent the result of ordinal regression. The p-value for each 2-sided test was adjusted using Benjamini-Hochberg false discovery rate correction (Adj. p).

Cohort	Effect	Comparison	Variable	Coefficient (95% CI)	SE	Unadjusted P	Adjusted P
UK Biobank	Male sex effect	46,XY:46,XX	Fluid Intelligence	0.11 (0.1- 0.12)	0.004	2.16E-157	2.16E-156
UK Biobank	Extra X effect	47,XXX:46,XX	Fluid Intelligence	-0.79 (-1.05- -0.53)	0.135	4.51E-09	1.13E-08
UK Biobank	Extra X effect	47,XXY:46,XY	Fluid Intelligence	-0.93 (-1.15- -0.7)	0.115	6.94E-16	3.47E-15
UK Biobank	Extra Y effect	47,XXY:46,XX	Fluid Intelligence	-0.81 (-1.02- -0.6)	0.108	4.12E-14	1.37E-13
UK Biobank	Extra Y effect	47,XYY:46,XY	Fluid Intelligence	-0.73 (-0.97- -0.48)	0.127	1.00E-08	2.00E-08
UK Biobank	Haploinsufficiency	45,X:46,XX	Fluid Intelligence	-0.13 (-0.57- 0.3)	0.221	5.48E-01	6.09E-01
UK Biobank	Haploinsufficiency	45,X:46,XY	Fluid Intelligence	-0.25 (-0.71- 0.22)	0.236	2.94E-01	3.68E-01
UK Biobank	Between SCAs	45,X:47,XXX	Fluid Intelligence	0.66 (0.27- 1.06)	0.201	1.01E-03	1.69E-03
UK Biobank	Between SCAs	47,XXY:47,XXX	Fluid Intelligence	-0.02 (-0.31- 0.27)	0.15	8.94E-01	8.94E-01
UK Biobank	Between SCAs	47,XYY:47,XXY	Fluid Intelligence	0.2 (-0.1- 0.5)	0.151	1.89E-01	2.70E-01
UK Biobank	Male sex effect	46,XY:46,XX	Pairs Matching	0.03 (0.03- 0.04)	0.003	3.09E-31	6.17E-30
UK Biobank	Extra X effect	47,XXX:46,XX	Pairs Matching	-0.16 (-0.35- 0.02)	0.094	8.18E-02	2.04E-01
UK Biobank	Extra X effect	47,XXY:46,XY	Pairs Matching	-0.12 (-0.26- 0.02)	0.071	8.91E-02	2.14E-01
UK Biobank	Extra Y effect	47,XXY:46,XX	Pairs Matching	-0.08 (-0.22- 0.05)	0.069	2.21E-01	4.42E-01
UK Biobank	Extra Y effect	47,XYY:46,XY	Pairs Matching	-0.17 (-0.33- 0)	0.086	5.61E-02	1.46E-01
UK Biobank	Haploinsufficiency	45,X:46,XX	Pairs Matching	-0.38 (-0.63- -0.13)	0.127	3.02E-03	1.13E-02
UK Biobank	Haploinsufficiency	45,X:46,XY	Pairs Matching	-0.41 (-0.67- -0.16)	0.132	1.65E-03	6.58E-03
UK Biobank	Between SCAs	45,X:47,XXX	Pairs Matching	-0.21 (-0.52- 0.09)	0.155	1.69E-01	3.62E-01
UK Biobank	Between SCAs	47,XXY:47,XXX	Pairs Matching	0.08 (-0.14- 0.3)	0.114	4.87E-01	6.36E-01
UK Biobank	Between SCAs	47,XYY:47,XXY	Pairs Matching	-0.04 (-0.24- 0.16)	0.102	6.63E-01	7.50E-01
UK Biobank	Male sex effect	46,XY:46,XX	Reaction Time	0.18 (0.18- 0.19)	0.003	0.00E+00	0.00E+00

UK Biobank	Extra X effect	47,XXX:46,XX	Reaction Time	-0.36 (-0.54- -0.19)	0.088	3.29E-05	1.64E-04
UK Biobank	Extra X effect	47,XXY:46,XY	Reaction Time	-0.44 (-0.57- -0.31)	0.066	2.73E-11	2.73E-10
UK Biobank	Extra Y effect	47,XXY:46,XX	Reaction Time	-0.26 (-0.39- -0.13)	0.064	4.93E-05	2.28E-04
UK Biobank	Extra Y effect	47,XYY:46,XY	Reaction Time	-0.38 (-0.54- -0.23)	0.081	2.16E-06	1.44E-05
UK Biobank	Haploinsufficiency	45,X:46,XX	Reaction Time	-0.53 (-0.76- -0.29)	0.12	1.18E-05	7.09E-05
UK Biobank	Haploinsufficiency	45,X:46,XY	Reaction Time	-0.7 (-0.95- -0.46)	0.123	1.17E-08	1.00E-07
UK Biobank	Between SCAs	45,X:47,XXX	Reaction Time	-0.16 (-0.51- 0.2)	0.181	3.90E-01	5.70E-01
UK Biobank	Between SCAs	47,XXY:47,XXX	Reaction Time	0.11 (-0.15- 0.36)	0.129	4.14E-01	5.92E-01
UK Biobank	Between SCAs	47,XYY:47,XXY	Reaction Time	0.05 (-0.17- 0.28)	0.114	6.36E-01	7.50E-01
UK Biobank	Male sex effect	46,XY:46,XX	Digit Span	0.15 (0.14- 0.16)	0.005	1.58E-194	4.75E-193
UK Biobank	Extra X effect	47,XXX:46,XX	Digit Span	-0.35 (-0.7- 0)	0.178	4.89E-02	1.38E-01
UK Biobank	Extra X effect	47,XXY:46,XY	Digit Span	-0.7 (-0.99- -0.42)	0.144	1.08E-06	8.08E-06
UK Biobank	Extra Y effect	47,XXY:46,XX	Digit Span	-0.55 (-0.84- -0.27)	0.144	1.21E-04	5.20E-04
UK Biobank	Extra Y effect	47,XYY:46,XY	Digit Span	-0.78 (-1.13- -0.42)	0.18	1.64E-05	8.92E-05
UK Biobank	Haploinsufficiency	45,X:46,XX	Digit Span	-0.24 (-0.92- 0.45)	0.35	4.96E-01	6.36E-01
UK Biobank	Haploinsufficiency	45,X:46,XY	Digit Span	-0.39 (-1.07- 0.3)	0.349	2.66E-01	4.84E-01
UK Biobank	Between SCAs	45,X:47,XXX	Digit Span	-0.07 (-0.89- 0.75)	0.418	8.66E-01	8.96E-01
UK Biobank	Between SCAs	47,XXY:47,XXX	Digit Span	-0.21 (-0.72- 0.31)	0.262	4.31E-01	6.01E-01
UK Biobank	Between SCAs	47,XYY:47,XXY	Digit Span	-0.1 (-0.61- 0.41)	0.262	7.00E-01	7.64E-01
UK Biobank	Male sex effect	46,XY:46,XX	Symbol Digit	-0.01 (-0.02- 0)	0.005	5.72E-03	2.02E-02
UK Biobank	Extra X effect	47,XXX:46,XX	Symbol Digit	-0.29 (-0.64- 0.07)	0.182	1.15E-01	2.66E-01
UK Biobank	Extra X effect	47,XXY:46,XY	Symbol Digit	-0.33 (-0.62- -0.03)	0.15	2.97E-02	8.92E-02
UK Biobank	Extra Y effect	47,XXY:46,XX	Symbol Digit	-0.35 (-0.65- -0.05)	0.154	2.20E-02	6.94E-02
UK Biobank	Extra Y effect	47,XYY:46,XY	Symbol Digit	-0.51 (-0.93- -0.08)	0.215	1.89E-02	6.28E-02
UK Biobank	Haploinsufficiency	45,X:46,XX	Symbol Digit	0.03 (-0.7- 0.76)	0.371	9.34E-01	9.34E-01
UK Biobank	Haploinsufficiency	45,X:46,XY	Symbol Digit	0.05 (-0.66- 0.76)	0.362	8.89E-01	9.04E-01
UK Biobank	Between SCAs	45,X:47,XXX	Symbol Digit	0.31 (-0.6- 1.21)	0.462	5.07E-01	6.36E-01
UK Biobank	Between SCAs	47,XXY:47,XXX	Symbol Digit	-0.06 (-0.6- 0.47)	0.273	8.16E-01	8.59E-01

UK Biobank	Between SCAs	47,XYY:47,XXY	Symbol Digit	-0.21 (-0.84- 0.41)	0.319	5.09E-01	6.36E-01
UK Biobank	Male sex effect	46,XY:46,XX	Trail Making 1	-0.04 (-0.05- -0.03)	0.006	5.93E-13	7.11E-12
UK Biobank	Extra X effect	47,XXX:46,XX	Trail Making 1	0.26 (-0.15- 0.66)	0.206	2.15E-01	4.42E-01
UK Biobank	Extra X effect	47,XXY:46,XY	Trail Making 1	0.21 (-0.14- 0.57)	0.183	2.43E-01	4.69E-01
UK Biobank	Extra Y effect	47,XXY:46,XX	Trail Making 1	0.17 (-0.16- 0.51)	0.171	3.06E-01	5.18E-01
UK Biobank	Extra Y effect	47,XYY:46,XY	Trail Making 1	0.51 (0- 1.01)	0.259	5.07E-02	1.38E-01
UK Biobank	Haploinsufficiency	45,X:46,XX	Trail Making 1	0.42 (-0.43- 1.27)	0.432	3.30E-01	5.21E-01
UK Biobank	Haploinsufficiency	45,X:46,XY	Trail Making 1	0.46 (-0.45- 1.37)	0.462	3.19E-01	5.18E-01
UK Biobank	Between SCAs	45,X:47,XXX	Trail Making 1	0.18 (-0.65- 1)	0.419	6.75E-01	7.50E-01
UK Biobank	Between SCAs	47,XXY:47,XXX	Trail Making 1	-0.09 (-0.64- 0.47)	0.283	7.55E-01	8.09E-01
UK Biobank	Between SCAs	47,XYY:47,XXY	Trail Making 1	0.32 (-0.26- 0.91)	0.299	2.80E-01	4.94E-01
UK Biobank	Male sex effect	46,XY:46,XX	Trail Making 2	-0.05 (-0.07- -0.04)	0.006	1.39E-20	2.08E-19
UK Biobank	Extra X effect	47,XXX:46,XX	Trail Making 2	0.23 (-0.18- 0.64)	0.207	2.66E-01	4.84E-01
UK Biobank	Extra X effect	47,XXY:46,XY	Trail Making 2	0.13 (-0.23- 0.49)	0.185	4.87E-01	6.36E-01
UK Biobank	Extra Y effect	47,XXY:46,XX	Trail Making 2	0.07 (-0.27- 0.42)	0.175	6.69E-01	7.50E-01
UK Biobank	Extra Y effect	47,XYY:46,XY	Trail Making 2	0.35 (-0.15- 0.86)	0.257	1.68E-01	3.62E-01
UK Biobank	Haploinsufficiency	45,X:46,XX	Trail Making 2	0.41 (-0.45- 1.26)	0.435	3.51E-01	5.39E-01
UK Biobank	Haploinsufficiency	45,X:46,XY	Trail Making 2	0.46 (-0.44- 1.36)	0.459	3.17E-01	5.18E-01
UK Biobank	Between SCAs	45,X:47,XXX	Trail Making 2	0.18 (-0.59- 0.95)	0.393	6.52E-01	7.50E-01
UK Biobank	Between SCAs	47,XXY:47,XXX	Trail Making 2	-0.17 (-0.7- 0.35)	0.268	5.24E-01	6.41E-01
UK Biobank	Between SCAs	47,XYY:47,XXY	Trail Making 2	0.26 (-0.31- 0.82)	0.288	3.70E-01	5.56E-01
UK Biobank	Male sex effect	46,XY:46,XX	Household Income	0.33 (0.32- 0.34)	0.006	0.00E+00	0.00E+00
UK Biobank	Extra X effect	47,XXX:46,XX	Household Income	-1.66 (-2.09- -1.24)	0.216	1.29E-14	3.23E-14
UK Biobank	Extra X effect	47,XXY:46,XY	Household Income	-1.62 (-1.9- -1.34)	0.144	2.33E-29	1.55E-28
UK Biobank	Extra Y effect	47,XXY:46,XX	Household Income	-1.34 (-1.62- -1.05)	0.145	3.04E-20	1.22E-19
UK Biobank	Extra Y effect	47,XYY:46,XY	Household Income	-1.54 (-1.89- -1.2)	0.177	2.15E-18	7.17E-18
UK Biobank	Haploinsufficiency	45,X:46,XX	Household Income	-0.73 (-1.26- -0.19)	0.273	7.67E-03	1.10E-02
UK Biobank	Haploinsufficiency	45,X:46,XY	Household Income	-1.03 (-1.56- -0.5)	0.27	1.44E-04	2.62E-04

UK Biobank	Between SCAs	45,X:47,XXX	Household Income	1.27 (0.56- 1.99)	0.366	5.08E-04	8.46E-04
UK Biobank	Between SCAs	47,XXY:47,XXX	Household Income	0.63 (0.11- 1.16)	0.267	1.76E-02	2.35E-02
UK Biobank	Between SCAs	47,XYY:47,XXY	Household Income	0.05 (-0.4- 0.5)	0.23	8.22E-01	8.22E-01
UK Biobank	Male sex effect	46,XY:46,XX	Educational attainment	-0.02 (-0.03- -0.01)	0.006	2.93E-03	4.51E-03
UK Biobank	Extra X effect	47,XXX:46,XX	Educational attainment	-1.18 (-1.52- -0.84)	0.173	8.08E-12	1.80E-11
UK Biobank	Extra X effect	47,XXY:46,XY	Educational attainment	-1.36 (-1.61- -1.11)	0.127	8.38E-27	4.19E-26
UK Biobank	Extra Y effect	47,XXY:46,XX	Educational attainment	-1.5 (-1.75- -1.25)	0.128	1.45E-31	1.45E-30
UK Biobank	Extra Y effect	47,XYY:46,XY	Educational attainment	-1.25 (-1.55- -0.95)	0.154	5.93E-16	1.69E-15
UK Biobank	Haploinsufficiency	45,X:46,XX	Educational attainment	0.37 (-0.13- 0.87)	0.254	1.46E-01	1.63E-01
UK Biobank	Haploinsufficiency	45,X:46,XY	Educational attainment	0.41 (-0.08- 0.89)	0.247	9.97E-02	1.17E-01
UK Biobank	Between SCAs	45,X:47,XXX	Educational attainment	1.73 (1.08- 2.38)	0.33	1.59E-07	3.17E-07
UK Biobank	Between SCAs	47,XXY:47,XXX	Educational attainment	-0.37 (-0.8- 0.07)	0.222	9.77E-02	1.17E-01
UK Biobank	Between SCAs	47,XYY:47,XXY	Educational attainment	0.13 (-0.27- 0.54)	0.205	5.11E-01	5.37E-01
All of Us	Male sex effect	46,XY:46,XX	Household Income	0.11 (0.09- 0.13)	0.01	8.10E-31	1.13E-29
All of Us	Extra X effect	47,XXX:46,XX	Household Income	-1.82 (-2.32- -1.33)	0.252	4.67E-13	1.09E-12
All of Us	Extra X effect	47,XXY:46,XY	Household Income	-1.63 (-2.03- -1.23)	0.202	7.50E-16	3.50E-15
All of Us	Extra Y effect	47,XXY:46,XX	Household Income	-1.68 (-2.09- -1.27)	0.208	6.10E-16	3.50E-15
All of Us	Extra Y effect	47,XYY:46,XY	Household Income	-1.83 (-2.36- -1.3)	0.269	1.05E-11	2.09E-11
All of Us	Between SCAs	47,XXY:47,XXX	Household Income	-0.09 (-0.76- 0.57)	0.339	7.87E-01	7.87E-01
All of Us	Between SCAs	47,XYY:47,XXY	Household Income	-0.16 (-0.83- 0.5)	0.338	6.26E-01	7.30E-01
All of Us	Male sex effect	46,XY:46,XX	Educational attainment	-0.02 (-0.04- 0)	0.009	4.93E-02	6.90E-02
All of Us	Extra X effect	47,XXX:46,XX	Educational attainment	-1.53 (-1.99- -1.08)	0.231	2.96E-11	5.18E-11
All of Us	Extra X effect	47,XXY:46,XY	Educational attainment	-1.4 (-1.77- -1.04)	0.186	5.64E-14	1.58E-13
All of Us	Extra Y effect	47,XXY:46,XX	Educational attainment	-1.54 (-1.92- -1.16)	0.192	1.02E-15	3.56E-15
All of Us	Extra Y effect	47,XYY:46,XY	Educational attainment	-1.59 (-2.08- -1.09)	0.253	3.53E-10	5.49E-10
All of Us	Between SCAs	47,XXY:47,XXX	Educational attainment	-0.11 (-0.74- 0.52)	0.321	7.40E-01	7.87E-01
All of Us	Between SCAs	47,XYY:47,XXY	Educational attainment	-0.2 (-0.83- 0.43)	0.321	5.26E-01	6.69E-01

SCA=sex chromosome aneuploidy; CI=confidence interval; SE=standard error

Table S12. ICD codes used to identify ASD, ID, and chromosomal anomaly diagnoses.

	ICD-9	ICD-10
Autism spectrum disorder	299	F84
	299.8	
	299.9	
Intellectual disability	317	F70
	318	F71
	318.1	F72
	318.2	F73
	319	F78
	315.5	F79
	315.8	F88
Chromosomal abnormality	758.6	Q96.9

	758.7	Q97.0
	758.81	Q97.1
	758.9	Q97.2
	759.9	Q97.8
		Q98.0
		Q98.4
		Q98.5
		Q98.7
		Q98.8
		Q98.9
		Q99.9

Table S13. ASD and ID counts in MyCode cohort.

Sex Chromosome Complement	ASD	ID	ASD or ID
46,XX	241	741	843
46,XY	701	991	1302
47,XXY	2	2	3
47,XYY	1	6	6
47,XXX	0	3	3
45,X	1	2	2

SCA=sex chromosome aneuploidy; ASD=autism spectrum disorder; ID=intellectual disabilities

Table S14. Identification of sex chromosome aneuploidy using genotype arrays.

Cohort	SPARK				MyCode				UK Biobank	All of Us
Batch No.	GSA Batch #1	GSA Batch #2	GSA Batch #3	GSA Batch #4	GSA Batch #1	GSA Batch #2	GSA Batch #3	OMNI	Axiom	Illumina
Sequencing platform	Illumina Global Screening Array GSA-24v1	Illumina Global Screening Array GSA-24v1	Illumina Global Screening Array GSA-24v1	Illumina Global Screening Array GSA-24v1	Illumina Global Screening Array GSA-24v1/GSA-24v2	Illumina Global Screening Array GSA-24v1/GSA-24v2	Illumina Global Screening Array GSA-24v1/GSA-24v2	OmniExpressExome-8 Kit	Affymetrix UK BiLEVE Axiom array and UK Biobank Axiom Array	Illumina Infinium Global Diversity Array
Individuals lost to genotype array QC*	4366	2108	1003	3619	4907	1238	4246	1441	NA	2811
47,XXX definition	chrX LRR median > 0.1 & chrY LRR median < -1.7	chrX LRR median > 0.1 & chrY LRR median < -1.7	chrX LRR median > 0.14 & chrY LRR median < -1.7	chrX LRR median > 0.15 & chrY LRR median < -1.7	chrX LRR median > 0.06 & chrY LRR median < -1.7	chrX LRR median > 0.1 & chrY LRR median < -1.7	chrX LRR median > 0.15 & chrY LRR median < -1.7	chrX LRR median > 0.15 & chrY LRR median < -1.7 & chrX LRR median ≤ 0.31	L2R Mean.y < -0.5 & L2R Mean.x > 0.15 & het BAF x sum < 40	mean chrY < -2 & mean chrX > 0.2 & het BAF x sum < 200
47,XXY definition	chrY LRR median ≥ -1.0 & chrY LRR median < .2 & chrX LRR median > -0.15 &	chrY LRR median ≥ -1.0 & chrY LRR median < .2 & chrX LRR median > -0.15 &	chrY LRR median ≥ -1.0 & chrY LRR median < .2 & chrX LRR median > -0.15 &	chrY LRR median ≥ -1.0 & chrY LRR median < .4 & chrX LRR median > -0.15 &	chrY LRR median ≥ -1.5 & chrX LRR median > -0.2 & chrX LRR median ≤ 0.05	chrY LRR median ≥ -1.5 & chrY LRR median < .1 & chrX LRR median > -0.15	chrY LRR median ≥ -1.0 & chrY LRR median < .2 & chrX LRR median > -0.15	chrY LRR median ≥ -1.5 & chrX LRR median > -0.2 & chrX LRR median ≤ 0.15	L2R Mean.y ≥ -0.5 & L2R Mean.y < 0.23 & L2R Mean.x > -0.22	mean chrY ≥ -2 & mean chrY < -0.3 & mean chrX > 0 & mean chrX < 0.2 OR mean chrY

	chrX LRR median < 0.1	chrX LRR median < 0.1	chrX LRR median < 0.1	chrX LRR median < 0.1						≥ -2 & mean chrY < -0.5 & mean chrX > -0.03 & mean chrX < 0.2
47,XYY definition	chrY LRR median ≥ 0.23 & chrX LRR median ≤ -0.2	chrY LRR median ≥ 0.23 & chrX LRR median ≤ -0.2	chrY LRR median ≥ 0.26 & chrX LRR median ≤ -0.4	chrY LRR median ≥ 0.4 & chrX LRR median ≤ -0.15	chrY LRR median ≥ -0.33 & chrX LRR median ≤ -0.2	chrY LRR median ≥ 0.3 & chrX LRR median ≤ -0.15	chrY LRR median ≥ 0.23 & chrX LRR median ≤ -0.37	chrY LRR median ≥ 0.14 & chrX LRR median ≤ -0.3	L2R Mean.y ≥ 0.22 & L2R Mean.x ≤ -0.17	mean chrY ≥ -0.3 & mean chrX ≤ -0.1 ~ "XYY"
45,X nonmosaic defintion	chrY LRR median < -2.5 & chrX LRR median < -0.26	chrY LRR median < -2.5 & chrX LRR median < -0.39	chrY LRR median < -2.5 & chrX LRR median < -0.36	chrY LRR median < -2 & chrX LRR median < -0.25	chrY LRR median < -2.5 & chrX LRR median < -0.24	chrY LRR median < -2.5 & chrX LRR median < -0.27	chrY LRR median < -2.5 & chrX LRR median < -0.32	chrY LRR median < -3.0 & chrX LRR median < -0.27	L2R Mean.y < -1.22 & L2R Mean.x < -0.30 & het BAF x sum < 40	mean chrY < -3 & mean chrX < -0.58 & het BAF x sum < 175
45,X mosaic defintion	chrY LRR median < -2.5 & chrX LRR median < -0.18 & chrX LRR median ≥ -0.26	chrY LRR median < -2.5 & chrX LRR median < -0.18 & chrX LRR median ≥ -0.39	chrY LRR median < -2.5 & chrX LRR median < -0.18 & chrX LRR median ≥ -0.36	chrY LRR median < -2 & chrX LRR median < -0.18 & chrX LRR median ≥ -0.25	No mosaic 45,X	chrY LRR median < -2.5 & chrX LRR median < -0.24 & chrX LRR median ≥ -0.27	chrY LRR median < -2.5 & chrX LRR median < -0.23 & chrX LRR median ≥ -0.32	chrY LRR median < -3.0 & chrX LRR median < -0.14 & chrX LRR median ≥ -0.27	L2R Mean.y < -1.1 & L2R Mean.x < -0.17 & L2R Mean.x ≥ -0.30	mean chrY < -3 & mean chrX < 0 & mean chrX ≥ -0.58 & het BAF x sum < 175

*For Illumina platforms, individuals with chromosome 1 Log R ratio (LRR) > 0.28 were removed. This filtering did not apply to UK Biobank participant data sequenced on the Axiom arrays, which underwent QC by the UK Biobank.

Table S15. Samples removed from each cohort during quality control.

SPARK	MyCode	UK Biobank	All of Us
4,383 removed after QC (Chr1 LRR SD > 0.28)	14,540 removed after QC (Chr1 LRR SD > 0.28)	195 sex mismatches removed	2,811 removed after QC (Chr1 LRR SD > 0.28)
4 sex mismatches removed	2 LRR outliers removed after failed sex chromosome-wide visual validation	Removed 82 individuals with excluded karyotypes (80 mosaic 45,X; 2 48,XXYY)	<20 LRR outliers removed after failed sex chromosome-wide visual validation*
Removed 8 individuals with excluded karyotypes (4 mosaic 45,X; 2 48,XXXY; 2 48,XXYY)	23 sex mismatches removed	33 ASD/ID cases removed	Removed 66 individuals with excluded karyotypes (mosaic 45,X; 48,XXYY; 48,XXXY)*
	Removed 29 individuals with excluded karyotypes (24 mosaic 45,X; 2 48,XXYY; 2 48,XXXY; 1 48,XXXX)		773 ASD/ID cases removed
	2,159 ASD/ID cases removed		

*For the All of Us cohort, no data or statistics can be reported that allow a participant count of 1 to 20 to be derived.

Supplementary References

1. Nagraj, V. P. twoxtwo: Work with Two-by-Two Tables. Preprint at https://CRAN.R-project.org/package=twoxtwo (2021).
2. Kirov, G. *et al.* The penetrance of copy number variations for schizophrenia and developmental delay. *Biol. Psychiatry* **75**, 378–385 (2014).
3. Vassos, E. *et al.* Penetrance for copy number variants associated with schizophrenia. *Hum. Mol. Genet.* **19**, 3477–3481 (2010).
4. Maenner, M. J. Prevalence and Characteristics of Autism Spectrum Disorder Among Children Aged 8 Years — Autism and Developmental Disabilities Monitoring Network, 11 Sites, United States, 2020. *MMWR Surveill. Summ.* **72**, (2023).
5. Kendall, K. M. *et al.* Cognitive performance and functional outcomes of carriers of pathogenic copy number variants: analysis of the UK Biobank. *Br. J. Psychiatry* 1–8 (2019).
6. Benjamini, Y. & Hochberg, Y. Controlling the false discovery rate: A practical and powerful approach to multiple testing. *J. R. Stat. Soc.* **57**, 289–300 (1995).
7. Viechtbauer, W. Conducting Meta-Analyses in R with the metafor Package. *J. Stat. Softw.* **36**, 1–48 (2010).
