# Supplementary material for: A genome-first study of sex chromosome aneuploidies provides evidence of Y chromosome dosage effects on autism risk
Source: Nat Commun. 2024 Oct 15;15:8897. doi: 10.1038/s41467-024-53211-7 (PMC11480344; doi:10.1038/s41467-024-53211-7)
Supplement: Supplementary file 3 — Reporting Summary [file 41467_2024_53211_MOESM3_ESM.pdf]

nature portfolio

Corresponding author(s): Matthew Oetjens

Last updated by author(s): Aug 7, 2024

Reporting Summary

Nature Portfolio wishes to improve the reproducibility of the work that we publish. This form provides structure for consistency and transparency in reporting. For further information on Nature Portfolio policies, see our Editorial Policies and the Editorial Policy Checklist.

Statistics

For all statistical analyses, confirm that the following items are present in the figure legend, table legend, main text, or Methods section.

n/a	Confirmed	
☐	☒	The exact sample size (*n*) for each experimental group/condition, given as a discrete number and unit of measurement
☐	☒	A statement on whether measurements were taken from distinct samples or whether the same sample was measured repeatedly
☐	☒	The statistical test(s) used AND whether they are one- or two-sided<br>*Only common tests should be described solely by name; describe more complex techniques in the Methods section.*
☐	☒	A description of all covariates tested
☐	☒	A description of any assumptions or corrections, such as tests of normality and adjustment for multiple comparisons
☐	☒	A full description of the statistical parameters including central tendency (e.g. means) or other basic estimates (e.g. regression coefficient) AND variation (e.g. standard deviation) or associated estimates of uncertainty (e.g. confidence intervals)
☐	☒	For null hypothesis testing, the test statistic (e.g. *F*, *t*, *r*) with confidence intervals, effect sizes, degrees of freedom and *P* value noted<br>*Give P values as exact values whenever suitable.*
☒	☐	For Bayesian analysis, information on the choice of priors and Markov chain Monte Carlo settings
☒	☐	For hierarchical and complex designs, identification of the appropriate level for tests and full reporting of outcomes
☐	☒	Estimates of effect sizes (e.g. Cohen's *d*, Pearson's *r*), indicating how they were calculated

Our web collection on statistics for biologists contains articles on many of the points above.

Software and code

Policy information about availability of computer code

Data collection	Custom R and python scripts were used to call and QC sex chromosome aneuploidies from genotype data.
Data analysis	R 4.3.2 code used for this study can be made by contacting our study team.

For manuscripts utilizing custom algorithms or software that are central to the research but not yet described in published literature, software must be made available to editors and reviewers. We strongly encourage code deposition in a community repository (e.g. GitHub). See the Nature Portfolio guidelines for submitting code & software for further information.

Data

Policy information about availability of data

All manuscripts must include a data availability statement. This statement should provide the following information, where applicable:

- Accession codes, unique identifiers, or web links for publicly available datasets
- A description of any restrictions on data availability
- For clinical datasets or third party data, please ensure that the statement adheres to our policy

All analyses reported in this manuscript were performed on existing genomic and phenotypic datasets. Researchers can request access to the SPARK genetic and phenotypic data through by SFARI Base at: https://base.sfari.org. Researchers can register to access the UK Biobank resource at: https://www.ukbiobank.ac.uk. All sequencing data used in this study are available on the All of Us Researcher Workbench in the v7 release. Researchers can register to access this resource at: https://www.researchallofus.org/. The MyCode SCA dataset can be made available by contacting the investigators directly.

Research involving human participants, their data, or biological material

Policy information about studies with human participants or human data. See also policy information about sex, gender (identity/presentation), and sexual orientation and race, ethnicity and racism.

Reporting on sex and gender	Researchers can obtain the SNP genotyping data SPARK cohort used in this study (https://www.sfari.org/resource/spark/) by applying at https://base.sfari.org. The UK Biobank SNP genotyping phenotypic and cognitive measures can be obtained by applying at the UK Biobank database (https://www.ukbiobank.ac.uk/). All genotype data used in this study are available on the All of Us Researcher Workbench in the v7 release. Researchers can register to access this resource at: https://www.researchallofus.org/. R code used for this study can be made by contacting our study team.
Reporting on race, ethnicity, or other socially relevant groupings	SDOH variables were not as a grouping variable but we examined as outcomes in the study. The primary analyses were not stratified by a race or ethnicity but we performed secondary analyses restricting the cohort to individuals of European ancestry. European ancestry was determined based on a principal components analysis of genotype data and clustering with indivdiuals from the 1000 genomes reference population.
Population characteristics	The SPARK study (Simons Foundation Powering Autism Research for Knowledge) is a dataset of individuals with a diagnosis of autism and their families to date to study the genetic underpinnings of autism in the United States. Initiated in 2015, SPARK involves collaboration among 31 university-affiliated research clinics in 26 states across the United States. The Geisinger MyCode Community Health Initiative is a large health care–based cohort with linked electronic health records that began consenting patients on February 8, 2007, MyCode recruits primarily adult patients. Patients were recruited into MyCode during a primary care or specialty clinic visit at Geisinger, and eligibility did not depend on a particular condition, diagnosis, or demographic characteristic. Previous studies have shown that MyCode is similar in clinical characteristics to the Geisinger adult patient population. The UK Biobank (UKB) is a large epidemiological cohort containing extensive self-reported health data with linkage to hospital inpatient records in addition to genotype array data for most participants. This study was conducted under UK Biobank project number 49945. All of Us aims to engage a longitudinal cohort of one million or more US participants, with a focus on including populations that have historically been under-represented in biomedical research. Details of the All of Us cohort have been described previously.
Recruitment	All participants were recruited to SPARK under a centralized Institutional Review Board (IRB) protocol (Western IRB Protocol #20151664) and provided written informed consent to take part in the study. MyCode recruits primarily adults from the Geisinger patient population. Informed consent was obtained from adult patients and from the parents or guardians of pediatric patients. All participants provided informed consent to participate in UK Biobank projects. Informed consent for all All of Us participants was conducted in person or through an eConsent platform that includes primary consent, HIPAA Authorization for Research use of EHRs and other external health data, and Consent for Return of Genomic Results. The protocol was reviewed by the IRB of the All of Us Research Program.
Ethics oversight	The Geisinger institutional review board approved the study. The UK Biobank has ethical approval from the North West Multi-Centre Ethics Committee. All participants were recruited to SPARK under a centralized Institutional Review Board (IRB) protocol (Western IRB Protocol #20151664). The All of Us protocol was reviewed by the IRB of the All of Us Research Program.

Note that full information on the approval of the study protocol must also be provided in the manuscript.

Field-specific reporting

Please select the one below that is the best fit for your research. If you are not sure, read the appropriate sections before making your selection.

☒ Life sciences ☐ Behavioural & social sciences ☐ Ecological, evolutionary & environmental sciences

For a reference copy of the document with all sections, see nature.com/documents/nr-reporting-summary-flat.pdf

Life sciences study design

All studies must disclose on these points even when the disclosure is negative.

Sample size	he study population for ASD case-control analyses, the SPARKMC-SCA cohort, included 25,085 cases from SPARK (19,590 males, 5,495 females) and 152,331 controls from MyCode (59,419 males, 92,912 females)
Data exclusions	MyCode, All of Us, and UKB participants with ASD or ID were removed to curate control cohorts, which secondarily eliminated overlap between SPARK participants and controls from MyCode and All of Us. Although ASD and ID are frequently comorbid26,27, the EHRs of dually-affected adults rarely document both diagnoses; we therefore excluded controls with either ASD or ID diagnosis codes to ensure an ASD-negative cohort.
Replication	The between SCA analyses (eg. 47,XXY vs. 47,XYY) of ASD were repeated in an reanalysis of data reported by the iPSYCH consortium.
Randomization	This was a retrospective study of existing data. Therefore, there were no experimental groups and no randomization was required.
Blinding	n/a

Reporting for specific materials, systems and methods

We require information from authors about some types of materials, experimental systems and methods used in many studies. Here, indicate whether each material, system or method listed is relevant to your study. If you are not sure if a list item applies to your research, read the appropriate section before selecting a response.

Materials & experimental systems

n/a	Involved in the study
☒	☐ Antibodies
☒	☐ Eukaryotic cell lines
☒	☐ Palaeontology and archaeology
☒	☐ Animals and other organisms
☒	☐ Clinical data
☒	☐ Dual use research of concern
☒	☐ Plants

Methods

n/a	Involved in the study
☒	☐ ChIP-seq
☒	☐ Flow cytometry
☒	☐ MRI-based neuroimaging

Plants

Seed stocks	N/A
Novel plant genotypes	N/A
Authentication	N/A
